# X-ray Crystallographic Structure of α-Helical Peptide Stabilized by Hydrocarbon Stapling at *i*,*i* + 1 Positions

**DOI:** 10.3390/ijms22105364

**Published:** 2021-05-19

**Authors:** Yui Makura, Atsushi Ueda, Takuma Kato, Akihiro Iyoshi, Mei Higuchi, Mitsunobu Doi, Masakazu Tanaka

**Affiliations:** 1 Graduate School of Biomedical Sciences, Nagasaki University, 1–14 Bunkyo-machi, Nagasaki 852-8521, Japan; bb55720006@ms.nagasaki-u.ac.jp (Y.M.); bb55621002@ms.nagasaki-u.ac.jp (A.I.); m.higuchi005@gmail.com (M.H.); 2 Faculty of Pharmacy, Osaka Medical and Pharmaceutical University, Osaka 569-1094, Japan; t.kato@gly.oups.ac.jp (T.K.); mitsunobu.doi@ompu.ac.jp (M.D.)

**Keywords:** peptide, α-helix, hydrocarbon stapling, ring-closing metathesis, *i*,*i* + 1 staple, X-ray structure

## Abstract

Hydrocarbon stapling is a useful tool for stabilizing the secondary structure of peptides. Among several methods, hydrocarbon stapling at *i*,*i* + 1 positions was not extensively studied, and their secondary structures are not clarified. In this study, we investigate *i*,*i* + 1 hydrocarbon stapling between *cis*-4-allyloxy-l-proline and various olefin-tethered amino acids. Depending on the ring size of the stapled side chains and structure of the olefin-tethered amino acids, *E-* or *Z*-selectivities were observed during the ring-closing metathesis reaction (*E*/*Z* was up to 8.5:1 for 17–14-membered rings and up to 1:20 for 13-membered rings). We performed X-ray crystallographic analysis of hydrocarbon stapled peptide at *i*,*i* + 1 positions. The X-ray crystallographic structure suggested that the *i*,*i* + 1 staple stabilizes the peptide secondary structure to the right-handed α-helix. These findings are especially important for short oligopeptides because the employed stapling method uses two minimal amino acid residues adjacent to each other.

## 1. Introduction

**Figure 1:**
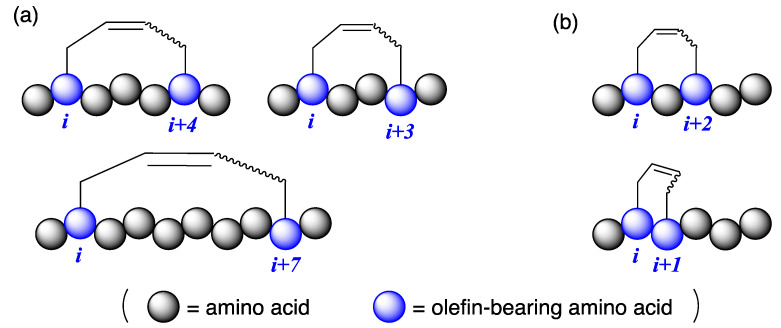
Peptides with hydrocarbon stapling at different positions. (**a**) Commonly used hydrocarbon stapling (at *i*,*i* + 4, *i*,*i* + 3, and *i*,*i* + 7), and (**b**) rarely investigated hydrocarbon stapling (at *i*,*i* + 2 and *i*,*i* + 1).

Introducing hydrocarbon stapling on the side chains of peptides is a promising technique for stabilizing the secondary structure of peptides and enhancing their functionalities [1,2,3,4,5]. Hydrocarbon stapling can be easily obtained by ring-closing metathesis reactions between olefin-bearing amino acid residues using Ru catalysts [6,7]. After the report on α-helicity-inducing all-hydrocarbon stapled peptides at *i*,*i* + 4 and *i*,*i* + 7 positions by Verdine et al. [8], several studies focused on the approach (as illustrated in Figure 1a) [9,10,11]. Currently, all-hydrocarbon stapled peptides are very important in drug development targeting protein–protein interactions because the pharmacophores interact via α-helical motifs [12]. Hydrocarbon stapling at *i*,*i* + 3 positions are reported in the literature [13,14,15]. For example, O’Leary et al. reported *E*-selective ring-closing metathesis between *O*-allyl-tethered l-serines at *i*,*i* + 3 positions to produce 3_10_-helical peptides [13]. Other hydrocarbon staples, such as *i*,*i* + 1 and *i*,*i* + 2, were not well researched, and their 3D structures are unknown (as illustrated in Figure 1b) [16,17,18,19]. In general, hydrocarbon stapling sacrifices two amino acid residues for the crosslinking motif, and those residues should not include essential residues for their biological activities. Based on this, the development of a large variety of hydrocarbon stapling at different positions can be achieved. Herein, we report hydrocarbon stapling of peptides at *i*,*i* + 1 positions by ring-closing metathesis reactions and the X-ray crystallographic structure of the right-handed α-helical octapeptide stabilized by *i*,*i* + 1 stapling.

## 2. Results and Discussion

Our previous report suggests the usefulness of cis-4-hydroxy-l-proline as an olefin-bearing amino acid for peptide stapling [19]. Thus, in this study, we started by optimizing the reaction conditions for *i*,*i* + 1 peptide stapling using cis-4-hydroxy-l-proline. We screened the ring-closing metathesis reaction at *i*,*i* + 1 positions using dipeptide **1** as the cyclization precursor (as illustrated in Table 1). The reaction catalyzed by 20 mol% of second-generation Grubbs catalyst in CH_2_Cl_2_ (20 mM) produced the desired **1′** in 55% yield as a mixture of *E*/*Z*-isomers (*E*/*Z* = 1.0:5.6; Entry 1). A comparable result was obtained using the first-generation Grubbs catalyst (Entry 2). Replacing the reaction solvents, such as toluene, 1,2-dichloroethane (DCE) and tetrahydrofuran (THF), decreased the yields and *Z*-selectivities (Entries 3–5). The reaction under diluted condition (5 mM in CH_2_Cl_2_) afforded the best yield at 76% (Entry 6). The reactions in refluxing CH_2_Cl_2_ resulted in insufficient yields due to the degradation of the desired product (Entries 8 and 9).

**Scheme 1:**
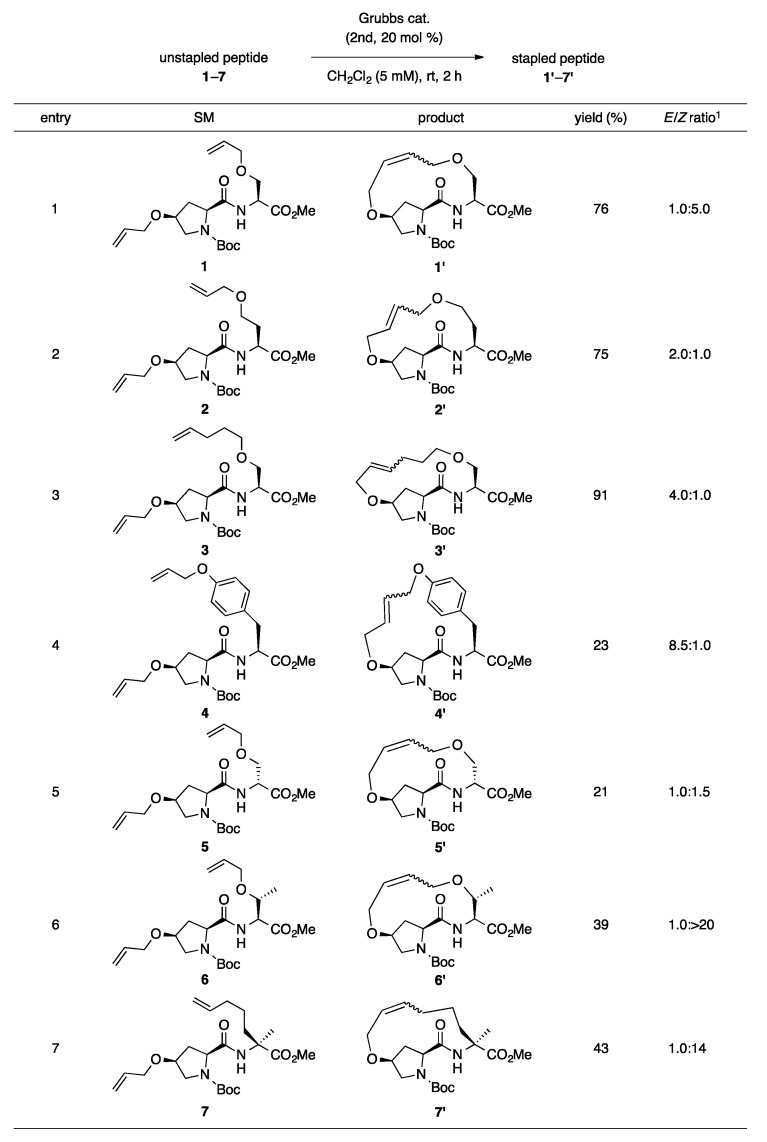
Substrate scope for ring-closing metathesis of peptides **1**–**7** at *i*,*i* + 1 positions. ^1^ Determined by ^1^H NMR.

Further, we investigated the substrate scope for the ring-closing metathesis of peptides at *i*,*i* + 1 positions using the optimized reaction conditions (as illustrated in Scheme 1). As the ring size of the stapled peptides increased from 13- to 15-membered rings, the yields and *E*-selectivities increased (Entries 1–3). l-Tyrosine and D-serine-derived unstapled peptides **4** and **5** produced the desired stapled peptides **4′** and **5′** in 23% and 21% yields, respectively, with large amounts of unreacted starting material (Entries 4 and 5). Surprisingly, high *Z*-selectivities were observed for the reaction of dipeptides **6** and **7**, which were composed of either *O*-allyl-tethered l-threonine or (*S*)-α-(4-pentenyl)alanine (Entries 6 and 7; *E*/*Z* = 1: >20 for **6′** and 1:14 for **7′**). These results suggest that α-methyl or β-methyl groups of *i* + 1 residue strongly affect the transition state of the ring-closing metathesis to yield *Z*-isomers.

**Scheme 2:**
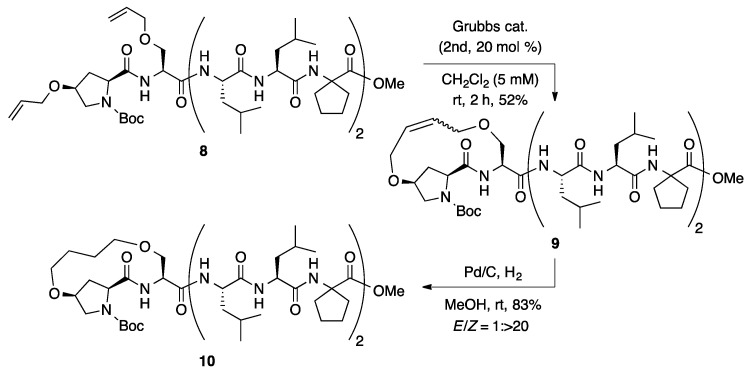
Ring-closing metathesis of octapeptide **8** at *i*,*i* + 1 positions.

The *i*,*i* + 1 hydrocarbon-stapling reaction of octapeptide **8**, in possession of 1-aminocycloalkane-1-carboxylic acid [20,21,22,23,24,25,26,27,28,29,30,31,32,33], was investigated under the optimized reaction conditions for the ring-closing metathesis (Scheme 2). In contrast with the moderate *Z*-selectivity of **1** (*E*/*Z* = 1:5), much higher Z-selectivity was observed for the ring-closing metathesis reaction of **8** (*E*/*Z* = 1: >20). The *Z*-selectivity could be influenced by their secondary structure. Hydrogenation of **9** afforded saturated stapled peptide **10** in high yield. The high *Z*-selectivities (*E*/*Z* was up to 1: >20) of the *i*,*i* + 1 hydrocarbon stapling is advantageous for peptide staples compared to those reported for *i*,*i* + 4 and *i*,*i* + 7 hydrocarbon stapling (*E*/*Z* was up to 1: >9) [15].

**Figure 2:**
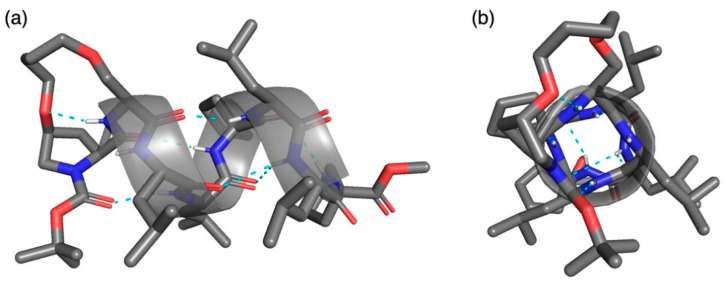
X-ray crystallographic structure of (*i*,*i* + 1)-stapled peptide **10**: a view (**a**) perpendicular to α-helical axis and (**b**) along helical axis from N-terminus.

Crystals suitable for X-ray crystallographic analyses were successfully obtained by slow evaporation of the solution of **10** in *N*,*N*-dimethylformamide (DMF)/water at room temperature (20–30 °C) [34]. The structure was solved in the orthorhombic *P*2_1_2_1_2_1_ space group to give an α-helical structure with a DMF molecule in the asymmetric unit (as illustrated in Figure 2 and Figure S1 and Table 2 and Table 3, and Table S1). To the best of our knowledge, this is the first X-ray crystallographic structure of α-helical stapled peptides at *i* and *i* + 1 positions. In the crystal state of the (*i*,*i* + 1)-stapled peptide **10**, four consecutive intramolecular hydrogen bonds of the *i*←*i* + 4 type, N(4)H···O = C(0) (N···O, 3.09 Å; N–H···O, 163.6°), N(5)H···O = C(1) (N···O, 2.98 Å; N–H···O, 168.6°), N(6)H···O = C(2) (N···O, 2.91 Å; N–H···O, 157.2°), and N(7)H···O = C(3) (N···O, 3.14 Å; N–H···O, 139.7°) were observed. These hydrogen bonds indicate the existence of the α-helical secondary structure in **10**. The average torsion angles of **10** at the N-terminus [avg.(ϕ1–ϕ5) = −62.4° and avg.(Ψ1–Ψ5) = −46.5°] were much closer to the ideal values of a right-handed α-helix [ϕ = −57° and Ψ = −47°] [35]. Therefore, the crosslinkage of the *i*,*i* + 1 staples at the N-terminus could affect the stabilization of the α-helical structure of **10**. On the C-terminus, weak intramolecular hydrogen bonds of the *i*←*i* + 3 type were observed, N(7)H···O = C(4) (N···O, 3.37 Å; N–H···O, 136.7°) and N(8)H···O = C(5) (N···O, 3.40 Å; N–H···O, 162.7°), while the N(8)–H···O(4) angle of *i*←*i* + 4 type was too small for a hydrogen bond. These bifurcated hydrogen bonds suggest that the conformation of the C-terminus exists as a mixture of α- and 3_10_-helix. Another intramolecular hydrogen bond between the N(2)–H of the main chain and ethereal oxygen of cis-4-hydroxyproline, N(2)H···O = C(Hyp^4^) (N···O, 2.93 Å; N–H···O, 137.6°), was observed. Such hydrogen bond stabilizes the secondary structures of peptides [30,36,37]. On the other hand, no intermolecular hydrogen bonds between peptides were observed in the packing mode (Figure S2). These results suggest that packing contacts have a small or no influence on the secondary structure of right-handed α-helix in this case. Thus, introducing hydrocarbon stapling at *i*,*i* + 1 positions using cis-4-hydroxyproline could be used for the stabilization of α-helical peptides likewise *i*,*i* + 4 and *i*,*i* + 7 staples. In our previous study, we reported asymmetric Michael addition of 1-methylindole to α,β-unsaturated aldehydes catalyzed by Boc-deprotected **10** [19]. We hypothesized that the reactive iminium ion intermediate between cis-4-hydroxy-l-proline and α,β-unsaturated aldehyde was formed inside the helical pipe with a rigid conformation caused by *i*,*i* + 1 staple. The X-ray crystallographic structure of **10** supports this observed conformation of the intermediate.

In summary, we developed *i*,*i* + 1 peptide stapling between cis-4-allyloxy-l-proline and various olefin-tethered amino acids. Depending on the ring size of the stapled peptides, *E*- or *Z*-selectivities were observed. The *E*-configured stapled product was preferred when the product was greater than a 14-membered ring, whereas the *Z*-configured isomer was preferred when the product was a 13-membered ring. The α-or β-methyl substituent of the *i* + 1 residue improved the *Z*-selectivities of the ring-closing metathesis (*E*:*Z* = 1: >20). X-ray crystallographic analysis of the octapeptide **10** revealed a stabilized α-helical structure. These results are useful for developing peptide-based organocatalysts [38,39,40] (i.e., considering mechanistic insights and structural modification of peptide catalysts based on the X-ray crystal structure), fluorinated peptides [41] (e.g., stabilization effects of using intramolecular hydrogen bonds beside main chain hydrogen bonds), and peptide-based drug delivery systems [42,43,44,45,46] (e.g., introducing *i*,*i* + 1 hydrocarbon stapling with essential residues for their biological activities remained intact).

## 3. Materials and Methods

### 3.1. General Procedure and Method

Melting points were taken on an AS ONE melting point apparatus ATM-01 (AS ONE Corporation, Osaka, Japan) and were uncorrected. Optical rotations were measured on a JASCO DIP-370 polarimeter (JASCO Corporation, Tokyo, Japan) using CHCl_3_ as a solvent. ^1^H NMR and ^13^C NMR spectra were recorded on the JEOL JNM-AL-400 (400 MHz), a Varian NMR System 500PS SN (500 MHz and 125 MHz) spectrometer (Agilent Inc., Santa Clara, CA, USA). Chemical shifts (δ) are reported in parts per million (ppm). For the ^1^H NMR spectra (CDCl_3_), tetramethylsilane was used as the internal reference (0.00 ppm), while the central solvent peak was used as the reference (77.0 ppm in CDCl_3_) for the ^13^C NMR spectra. The IR spectra were recorded on a Shimadzu IRAffinity-1 FT-IR spectrophotometer (Shimadzu Corporation, Kyoto, Japan). High-resolution mass spectra (HRMS) were obtained on a JEOL JMS-T100TD using electrospray ionization (ESI) (JEOL Ltd., Tokyo, Japan) or direct analysis in the realtime (DART) ionization in time-of-flight TOF mode. Analytical and semipreparative thin layer chromatography (TLC) was performed with Merck Millipore precoated TLC plates (MilliporeSigma, Burlington, VT, USA), silica gel 60 F_254_, and layer thicknesses of 0.25 and 0.50 mm, respectively. Compounds were observed in UV light at 254 nm and then visualized by staining with iodine, *p-*anisaldehyde, or phosphomolybdic acid stain. Flash and gravity column chromatography separations were performed on Kanto Chemical silica gel 60N, spherical neutral, with particle sizes of 63–210 μm and 40–50 μm, respectively. All moisture-sensitive reactions were conducted under an inert atmosphere. Reagents and solvents were of commercial grade and were used as supplied, unless otherwise noted. Compounds **1**, **8** [19], **S-1** [47,48], **S-2** [49,50], **S-3** [51], and **S-5** [52] were prepared according to the reported procedures. Copies of NMR Spectra are given in the Supplementary Materials.

### 3.2. Synthesis of Unstapled Dipeptides ***2***–***7***


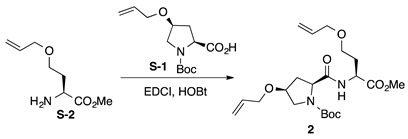


*Boc-l-Hyp^OAll^-l-Hse^OAll^-OMe* (**2**): to a solution of *N*-*tert*-butoxycarbonyl 4-*O*-allyl-*cis*-4-hydroxy-l-proline (Boc-L-Hyp^OAll^-OH, **S-1** [47,48]; 88.1 mg, 0.325 mmol) in CH_2_Cl_2_ (2 mL) were added *N*-(3-dimethylaminopropyl)-*N’*-ethylcarbodiimide hydrochloride (EDCI·HCl, 67.9 mg, 0.354 mmol) and 1-hydroxybenzotriazole hydrate (HOBt·H_2_O; 54.2 mg, 0.354 mmol) at 0 °C, and the solution was stirred for 30 min at 0 °C. Then, a solution of *O*-allyl-l-homoserine methyl ester (H-l-Hse^OAll^-OMe, **S-2** [49,50], 51.1 mg, 0.295 mmol) in CH_2_Cl_2_ (1 mL) was added to the reaction mixture at the same temperature, and the resultant mixture was gradually warmed to room temperature. After stirring for three days, CH_2_Cl_2_ was removed, and the residue was diluted with EtOAc. The solution was washed successively with 1 M of HCl, water, sat. aq NaHCO_3_, and brine. The organic layer was dried over anhydrous Na_2_SO_4_ and concentrated in vacuo to give a crude product, which was purified by flash column chromatography on silica gel (40% EtOAc in *n*-hexane) to give **2** (72.1 mg, 58%) as a pale yellow oil. *R*_f_ = 0.58 (EtOAc). ${\left[\alpha \right]}_{D}^{20}$ –11.0 (*c* 1.00, CHCl_3_). ^1^H NMR (500 MHz, CDCl_3_) δ: 7.38–7.17 (m, 1H), 5.98–5.76 (m, 2H), 5.34–5.09 (m, 4H), 4.72–4.56 (m, 1H), 4.42–4.25 (m, 1H), 4.11–4.05 (m, 1H), 4.05–3.84 (m, 4H), 3.73 (s, 0.6H), 3.72 (s, 2.4H), 3.63–3.39 (m, 4H), 2.65–2.41 (m, 1H), 2.27–1.96 (m, 3H), 1.48 (s, 9H). ^13^C NMR (125 MHz, CDCl_3_) δ: 172.5, 172.0, 171.0, 154.7, 134.5, 134.4, 134.34, 134.26, 117.3, 117.2, 117.1, 117.0, 80.9, 76.3, 72.04, 71.98, 69.6, 66.3, 66.0, 60.1, 52.7, 52.3, 52.1, 50.6, 50.4, 36.9, 35.6, 31.6, 28.3, 28.1. IR (film): 3385 (br), 2978, 2868, 1744, 1690 cm^−1^. HRMS (ESI) *m*/*z*: [M + Na]^+^ calcd. for C_21_H_34_N_2_O_7_Na, 449.2264; found, 449.2262.


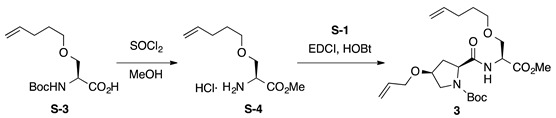


*Boc-l-Hyp^OAll^-l-Ser^OPte^-OMe* (**3**): to a solution of carboxylic acid **S-3** [51] (135 mg, 0.495 mmol) in MeOH (5 mL), thionyl chloride (0.143 mL, 1.98 mmol) was added dropwise at 0 °C. The reaction mixture was stirred at room temperature for 2 h and was concentrated to give H-L-Ser^OPte^-OMe·HCl (**S-4**, *R*_f_ = 0.57 with 0.5% AcOH in EtOAc), which was used for the next step without further purification. To a mixture of H-l-Ser^OPte^-OMe·HCl (**S-4**, 0.495 mmol) and Boc-l-Hyp^OAll^-OH (**S-1**, 148 mg, 0.545 mmol) in CH_2_Cl_2_ (5 mL) were added EDCI·HCl (114 mg, 0.594 mmol), HOBt·H_2_O (91.0 mg, 0.594 mmol), and DIPEA (0.253 mL, 1.49 mmol) at 0 °C, and the mixture was gradually warmed to room temperature. After stirring for 17 h, CH_2_Cl_2_ was removed under vacuum, and the residue was diluted with EtOAc. The resultant solution was washed successively with 1 M of HCl, water, sat. aq NaHCO_3_, and brine. The organic layer was dried over anhydrous Na_2_SO_4_ and concentrated in vacuo to give a crude product, which was purified by flash column chromatography on silica gel (40% EtOAc in *n*-hexane) to give **3** (90.1 mg, 41% in 2 steps) as a pale yellow oil. *R*_f_ = 0.71 (EtOAc). ${\left[\alpha \right]}_{D}^{22}$ –2.6 (*c* 1.00, CHCl_3_). ^1^H NMR (500 MHz, CDCl_3_) δ: 7.29 (br s, 1H), 7.11–6.90 (m, 1H), 5.98–5.71 (m, 2H), 5.34–5.22 (m, 1H), 5.21–5.11 (m, 1H), 5.05–4.92 (m, 2H), 4.77–4.63 (m, 1H), 4.44–4.28 (m, 1H), 4.11–3.92 (m, 2H), 3.92–3.78 (m, 2H), 3.75 (s, 3H), 3.66–3.48 (m, 3H), 3.47–3.35 (m, 2H), 2.67–2.45 (m, 1H), 2.24–2.02 (m, 3H), 1.67–1.57 (m, 2H), 1.49 (s, 9H). ^13^C NMR (125 MHz, CDCl_3_) δ: 172.1, 171.2, 170.7, 170.4, 154.7, 138.0, 134.4, 134.2, 117.2, 116.9, 114.79, 114.75, 81.0, 76.1, 72.0, 70.7, 70.6, 70.31, 70.27, 69.4, 65.9, 60.0, 52.8, 52.51, 52.45, 52.39, 52.2, 36.8, 35.3, 30.01, 29.99, 28.4, 28.2, 28.1. IR (film): 3428 (br), 2978, 2918, 1753, 1692 cm^−1^. HRMS (ESI) *m*/*z*: [M + Na]^+^ calcd. for C_22_H_36_N_2_O_7_Na, 463.2420; found, 463.2418.


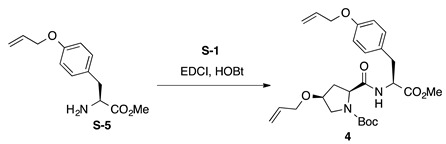


*Boc-l-Hyp^OAll^-l-Tyr^OAll^-OMe* (**4**): to a solution of Boc-L-Hyp^OAll^-OH (**S-1**, 445 mg, 1.64 mmol) in CH_2_Cl_2_ (8 mL) were added EDCI·HCl (314 mg, 1.64 mmol) and HOBt·H_2_O (301 mg, 1.97 mmol) at 0 °C, and the reaction mixture was stirred for 30 min at 0 °C. Then, a solution of *O*-allyl-l-tyrosine methyl ester (H-l-Tyr^OAll^-OMe, **S-5** [52], 386 mg, 1.64 mmol) in CH_2_Cl_2_ (3 mL) was added to the reaction mixture at the same temperature, and the resultant mixture was gradually warmed to room temperature. After stirring for 35 h, CH_2_Cl_2_ was removed in vacuo, and the residue was diluted with EtOAc. The resultant solution was washed successively with 1 M of HCl, water, sat. aq NaHCO_3_, and brine. The organic layer was dried over anhydrous Na_2_SO_4_ and concentrated in vacuo to give a crude product, which was purified by flash column chromatography on silica gel (50% EtOAc in *n*-hexane) to give **4** (562 mg, 70%) as a pale yellow oil. *R*_f_ = 0.75 (EtOAc). ${\left[\alpha \right]}_{D}^{23}$ +0.90 (*c* 1.00, CHCl_3_). ^1^H NMR (500 MHz, CDCl_3_) δ: 7.10–6.98 (m, 2H), 6.87–6.70 (m, 3H), 6.10–5.99 (m, 1H), 5.90–5.77 (m, 1H), 5.40 (dp, *J* = 17.2, 1.7 Hz, 1H), 5.31–5.20 (m, 2H), 5.19–5.10 (m, 1H), 4.89–4.76 (m, 1H), 4.54–4.45 (m, 2H), 4.42–4.21 (m, 1H), 4.12–4.02 (m, 1H), 4.00–3.91 (m, 1H), 3.91–3.83 (m, 1H), 3.65 (s, 3H), 3.55 (br s, 2H), 3.13–2.99 (m, 1H), 2.94 (br s, 1H), 2.53–2.41 (m, 1H), 2.21–1.95 (m, 1H), 1.38 (s, 9H). ^13^C NMR (125 MHz, CDCl_3_) δ: 171.9, 171.6, 171.5, 171.1, 171.0, 157.6, 155.4, 154.5, 134.3, 134.1, 133.22, 133.18, 130.5, 130.2, 128.0, 127.8, 117.6, 117.5, 117.3, 117.2, 114.7, 114.6, 114.4, 81.0, 76.1, 72.0, 69.5, 68.69, 68.67, 65.9, 60.1, 59.3, 53.7, 53.3, 53.1, 52.9, 52.2, 52.0, 37.3, 37.2, 36.9, 36.8, 35.0, 32.5, 28.2, 28.0. IR (film): 3424 (br), 2978, 2934, 1744, 1665 cm^−1^. HRMS (ESI) *m*/*z*: [M + Na]^+^ calcd. for C_26_H_36_N_2_O_7_Na, 511.2420; found, 511.2422.


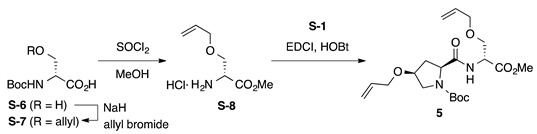


*Boc-l-Hyp^OAll^-d-Ser^OAll^-OMe* (**5**): to a solution of Boc-d-Ser-OH (**S-6**, 2.05 g, 10.0 mmol) in DMF (35 mL) was added sodium hydride (60% in mineral oil, 880 mg, 22.0 mmol) portionwise at −15 °C, and the reaction mixture was stirred at the same temperature for 2 h. To the above suspension, allyl bromide (0.952 mL, 11.0 mmol) was added dropwise at −15 °C, and the reaction mixture was stirred at room temperature for 14 h. The reaction mixture was quenched by adding water and washed twice with Et_2_O. The aqueous phase was acidified with 1 M of HCl, which was extracted with EtOAc three times. The combined organic layers were washed with water and brine, dried over anhydrous Na_2_SO_4,_ and concentrated under vacuum. The residue was purified by flash column chromatography on silica gel (40% EtOAc in *n*-hexane) to give Boc-d-Ser^OAll^-OH (**S-7**, 1.68 g, 69%, *R*_f_ = 0.28 with 10% MeOH in EtOAc) as a pale yellow oil. To a solution of **S-7** (123 mg, 0.500 mmol) in MeOH (5 mL) was added thionyl chloride (0.145 mL, 2.00 mmol) dropwise at 0 °C. The reaction mixture was stirred at room temperature for 2 h and was concentrated under vacuum to give crude H-d-Ser^OAll^-OMe·HCl (**S-8**, *R*_f_ = 0.57 with 0.5% AcOH in EtOAc), which was used for the next step without further purification. To a mixture of H-d-Ser^OAll^-OMe·HCl (**S-8**, 0.500 mmol) and Boc-l-Hyp^OAll^-OH (**S-1**, 149 mg, 0.550 mmol) in CH_2_Cl_2_ (5 mL) were added EDCI·HCl (115 mg, 0.600 mmol), HOBt·H_2_O (91.9 mg, 0.600 mmol), and DIPEA (0.255 mL, 1.50 mmol) at 0 °C, and the reaction mixture was gradually warmed to room temperature. After stirring for 17 h, CH_2_Cl_2_ was removed under vacuum, and the residue was diluted with EtOAc. The organic solution was washed successively with 1 M of HCl, water, sat. aq NaHCO_3_, and brine. The organic layer was dried over anhydrous Na_2_SO_4_ and concentrated in vacuo to give a crude product, which was purified by flash column chromatography on silica gel (40% EtOAc in *n*-hexane) to give **5** (84.6 mg, 41% in 2 steps) as a pale yellow oil. *R*_f_ = 0.66 (EtOAc). ${\left[\alpha \right]}_{D}^{23}$ –22.6 (*c* 1.00, CHCl_3_). ^1^H NMR (500 MHz, CDCl_3_) δ: 7.43–7.06 (m, 1H), 5.99–5.75 (m, 2H), 5.35–5.08 (m, 4H), 4.80–4.60 (m, 1H), 4.42–4.22 (m, 1H), 4.14–3.81 (m, 6H), 3.75 (s, 3H), 3.72–3.41 (m, 3H), 2.60–2.36 (m, 1H), 2.32–2.07 (m, 1H), 1.65–1.24 (m, 9H). ^13^C NMR (125 MHz, CDCl_3_) δ: 172.6, 171.2, 170.7, 170.3, 154.6, 134.4, 134.2, 134.0, 133.9, 117.4, 117.3, 117.2, 116.9, 80.7, 75.8, 72.14, 72.12, 72.0, 69.61, 69.59, 69.4, 65.9, 60.3, 59.7, 53.2, 52.6, 52.52, 52.46, 52.3, 36.8, 35.2, 33.4, 28.2. IR (film): 3325 (br), 2978, 2932, 1753, 1692 cm^−1^. HRMS (ESI) *m*/*z*: [M + Na]^+^ calcd. for C_20_H_32_N_2_O_7_Na, 435.2107; found, 435.2106.


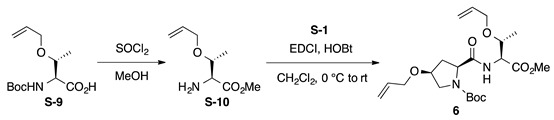


*Boc-l-Hyp^OAll^-l-Thr^OAll^-OMe* (**6**): to a solution of *N*-*tert*-butoxycarbonyl *O*-allyl-l-threonine (Boc-l-Thr^OAll^-OH, **S-9**; 130 mg, 0.500 mmol) in MeOH (2.5 mL) was added thionyl chloride (0.144 mL, 2.00 mmol) dropwise at 0 °C. The reaction mixture was stirred at room temperature for 3 h prior to the addition of sat. NaHCO_3_ aq. After removal of MeOH by evaporation, the aqueous residue was extracted with CHCl_3_ (five times) and the combined organics were dried over anhydrous Na_2_SO_4_. Concentration of the solution gave H-L-Thr^OAll^-OMe (**S-10**, 41.9 mg, 48%), which was used for the next step without further purification. To a solution of Boc-l-Hyp^OAll^-OH (**S-1**, 72.1 mg, 0.266 mmol) in CH_2_Cl_2_ (0.8 mL) were added EDCI·HCl (51.0 mg, 0.266 mmol) and HOBt·H_2_O (48.2 mg, 0.315 mmol) at 0 °C, and the solution was stirred for 30 min at 0 °C. Then, a solution of H-l-Thr^OAll^-OMe (**S-10**, 41.9 mg, 0.242 mmol) in CH_2_Cl_2_ (0.8 mL) was added to the reaction mixture at the same temperature, and the resultant mixture was gradually warmed to room temperature. After stirring for 42 h, CH_2_Cl_2_ was removed, and the residue was diluted with EtOAc. The solution was washed successively with 1 M of HCl, water, sat. aq NaHCO_3_, and brine. The organic layer was dried over anhydrous Na_2_SO_4_ and concentrated in vacuo to give a crude product, which was purified by flash column chromatography on silica gel (40% EtOAc in *n*-hexane) to give **6** (57.6 mg, 56%) as a pale yellow oil. *R*_f_ = 0.58 (EtOAc). ${\left[\alpha \right]}_{D}^{23}$ –11.2 (*c* 1.00, CHCl_3_). ^1^H NMR (400 MHz, CDCl_3_) δ: 6.92 (s, 1H), 5.98–5.70 (m, 2H), 5.35–5.05 (m, 4H), 4.71–4.55 (m, 1H), 4.45–4.29 (m, 1H), 4.15–3.79 (m, 6H), 3.74 (s, 0.6H), 3.73 (s, 2.4H), 3.63–3.46 (m, 2H), 2.70–2.40 (m, 1H), 2.30–2.10 (m, 1H), 1.49 (s, 9H), 1.18 (d, *J* = 6.3 Hz, 0.6H), 1.13 (d, *J* = 6.4 Hz, 2.4H). ^13^C NMR (100 MHz, CDCl_3_) δ: 172.5, 171.8, 171.1, 170.6, 154.9, 134.5, 134.2, 117.2, 117.0, 116.8, 81.0, 76.0, 74.4, 74.2, 72.0, 69.8, 69.7, 66.0, 60.2, 56.4, 52.7, 52.2, 52.1, 36.9, 35.6, 28.1, 16.3, 16.1. IR (film): 3441 (br), 2978, 2934, 1753, 1703 cm^−1^. HRMS (DART) *m*/*z*: [M + H]^+^ calcd. for C_21_H_35_N_2_O_7_, 427.2444; found, 427.2437.


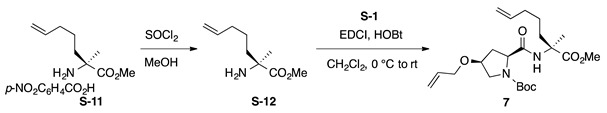


*Boc-l-Hyp^OAll^-(S)-Ala(4-Pte)-OMe* (**7**): to a solution of *p*-nitrobenzoic acid salt of (*S*)-(4-pentenyl)alanine *tert*-butyl ester (H-(*S*)-Ala(4-Pte)-O*^t^*Bu·*p*-NO_2_C_6_H_4_CO_2_H, **S-11**; 100 mg, 0.263 mmol) in MeOH (3 mL) was added thionyl chloride (0.152 mL, 2.10 mmol) dropwise at 0 °C. The reaction mixture was stirred at 65 °C for 69 h prior to the addition of sat. NaHCO_3_ aq. After removal of MeOH by evaporation, the aqueous residue was extracted with CHCl_3_ (five times) and the combined organics were dried over anhydrous Na_2_SO_4_. Concentration of the solution gave H-(*S*)-Ala(4-Pte)-OMe (**S-12**) contaminated with *p*-NO_2_C_6_H_4_CO_2_Me, which was used for the next step without further purification. To a solution of Boc-l-Hyp^OAll^-OH (**S-1**, 60.5 mg, 0.223 mmol) in CH_2_Cl_2_ (1.5 mL) were added EDCI·HCl (42.8 mg, 0.223 mmol) and HOBt·H_2_O (40.4 mg, 0.264 mmol) at 0 °C, and the solution was stirred for 30 min at 0 °C. Then, a solution of H-(*S*)-Ala(4-Pte)-OMe (**S-12**) in CH_2_Cl_2_ (0.5 mL) was added to the reaction mixture at the same temperature, and the resultant mixture was gradually warmed to room temperature. After stirring at room temperature for 3 d, CH_2_Cl_2_ was removed, and the residue was diluted with EtOAc. The solution was washed successively with 1 M of HCl, water, sat. aq NaHCO_3_, and brine. The organic layer was dried over anhydrous Na_2_SO_4_ and concentrated in vacuo to give a crude product, which was purified by flash column chromatography on silica gel (40% EtOAc in *n*-hexane) to give **7** (46.4 mg, 42% in 2 steps) as a pale yellow oil. *R*_f_ = 0.58 (EtOAc). ${\left[\alpha \right]}_{D}^{23}$ –16.2 (*c* 1.00, CHCl_3_). ^1^H NMR (500 MHz, CDCl_3_) δ: 7.28–6.82 (m, 1H), 5.99–5.79 (m, 1H), 5.79–5.67 (m, 1H), 5.35–5.22 (m, 1H), 5.22–5.13 (m, 1H), 5.03–4.90 (m, 2H), 4.33–4.13 (m, 1H), 4.10–3.84 (m, 3H), 3.77–3.71 (m, 3H), 3.71–3.61 (m, 1H), 3.56–3.46 (m, 1H), 2.50–2.32 (m, 1H), 2.30–2.06 (m, 2H), 2.05–1.98 (m, 2H), 1.83–1.74 (m, 1H), 1.58 (s, 1H), 1.53 (s, 2H), 1.48 (s, 9H), 1.43–1.34 (m, 1H), 1.23–1.13 (m, 1H). ^13^C NMR (125 MHz, CDCl_3_) δ: 174.8, 174.4, 171.4, 170.6, 154.9, 138.11, 138.06, 134.4, 134.2, 117.31, 117.25, 114.9, 114.8, 80.8, 80.6, 76.1, 72.1, 69.7, 66.2, 60.7, 60.1, 59.7, 52.9, 52.6, 52.4, 37.6, 36.5, 36.1, 35.5, 33.5, 33.4, 28.2, 23.4, 23.1, 23.0, 22.6. IR (film): 3393 (br), 2978, 2936, 1740, 1692 cm^−1^. HRMS (DART) *m*/*z*: [M + H]^+^ calcd. for C_22_H_37_N_2_O_6_, 425.2652; found, 425.2651.

### 3.3. Synthesis of Stapled Dipeptides ***1*′**–***7*′**

*Boc-l-Hyp^OX^-l-Ser^OX^-OMe* (**1′**; X = *n*-but-2-enyl tether): to a solution of unstapled peptide **1** [20] (20.5 mg, 0.0500 mmol) in degassed CH_2_Cl_2_ (10 mL) was added second-generation Grubbs catalyst (8.5 mg, 0.010 mmol) at room temperature under an argon atmosphere. The reaction mixture was stirred at the same temperature for 2 h and then passed through a short plug of amino silica gel/silica gel, which was eluted with EtOAc. After removal of the solvent, the residue was purified by flash column chromatography on silica gel (70% EtOAc in *n*-hexane) to give **1′** (14.6 mg, 76%) as a colorless oil. *R*_f_ = 0.32 (EtOAc). ^1^H NMR (500 MHz, CDCl_3_) δ: 7.23–7.00 (m, 1H), 5.86–5.73 (m, 1H), 5.68 (dt, *J* = 11.9, 6.5 Hz, 1H), 4.87–4.57 (m, 1H), 4.45–4.15 (m, 2H), 4.10–3.78 (m, 6H), 3.76 (s, 3H), 3.72–3.62 (m, 1H), 3.45 (dd, *J* = 12.1, 3.9 Hz, 1H), 2.66–2.47 (m, 1H), 2.26–2.11 (m, 1H), 1.60–1.39 (m, 9H). ^13^C NMR (125 MHz, CDCl_3_) δ: 172.2, 171.4, 170.2, 154.9, 130.4, 130.0, 129.4, 81.0, 80.8, 78.6, 77.9, 77.3, 67.7, 67.2, 66.4, 66.0, 65.1, 60.2, 59.8, 54.0, 53.2, 52.5, 52.5, 35.7, 34.2, 30.9, 29.7, 28.2. HRMS (DART) *m*/*z*: [M + H]^+^ calcd. for C_18_H_29_N_2_O_7_, 385.1975; found, 385.1970.

*Boc-l-Hyp^OX^-l-Hse^OX^-OMe* (**2′**; X = *n*-but-2-enyl tether): compound **2′** (15.0 mg, 75%) was obtained from compound **2** (21.3 mg, 0.0500 mmol) in a similar manner to that described for the synthesis of **1′**. Colorless oil. Eluent for column: 70% EtOAc/*n*-hexane. *R*_f_ = 0.34 (EtOAc). ^1^H NMR (500 MHz, CDCl_3_) δ: 6.78 (s, 1/3H), 6.41 (s, 2/3H), 5.86 (ddd, *J* = 10.8, 8.6, 6.6 Hz, 1/3H), 5.75 (dt, *J* = 15.7, 5.9 Hz, 2/3H), 5.74–5.59 (m, 1H), 4.78–4.66 (m, 1H), 4.35 (d, *J* = 10.0 Hz, 1H), 4.24–3.95 (m, 3H), 3.89–3.74 (m, 2H), 3.73 (s, 1H), 3.72 (s, 2H), 3.69–3.58 (m, 1H), 3.57–3.36 (m, 3H), 2.28–1.99 (m, 2H), 1.87–1.64 (m, 2H), 1.49 (s, 9H). ^13^C NMR (125 MHz, CDCl_3_) δ: 172.8, 172.2, 131.9, 131.7, 131.3, 128.7, 81.2, 75.9, 69.5, 68.5, 66.4, 63.5, 60.6, 60.2, 53.6, 52.3, 52.2, 49.4, 48.8, 34.7, 32.3, 28.2. HRMS (ESI) *m*/*z*: [M + Na]^+^ calcd. for C_19_H_30_N_2_O_7_Na, 421.1951; found, 421.1954.

*Boc-l-Hyp^OX^-l-Ser^OX^-OMe* (**3′**; X = *n*-hex-2-enyl tether): compound **3′** (18.8 mg, 91%) was obtained from compound **3** (22.0 mg, 0.0500 mmol) in a manner similar to that described for the synthesis of **1′**. Colorless oil. Eluent for column: 60% EtOAc/*n*-hexane. *R*_f_ = 0.42 (EtOAc). ^1^H NMR (500 MHz, CDCl_3_) δ: 7.85 (d, *J* = 8.1 Hz, 0.2H), 7.26–7.09 (m, 0.8H), 5.93–5.81 (m, 0.8H), 5.66 (dt, *J* = 14.9, 5.9 Hz, 0.1H), 5.60 (ddd, *J* = 9.1, 7.4, 6.2 Hz, 0.2H), 5.47 (dt, *J* = 15.3, 4.2 Hz, 0.7H), 5.42 (td, *J* = 10.1, 10.0, 5.1 Hz, 0.2H), 4.83–4.64 (m, 1H), 4.43–4.24 (m, 1H), 4.12 (t, *J* = 4.4 Hz, 0.2H), 4.04 (t, *J* = 3.8 Hz, 0.8H), 3.92–3.77 (m, 3H), 3.76 (s, 0.6H), 3.74 (s, 2.4H), 3.72–3.53 (m, 3H), 3.46–3.36 (m, 1H), 3.30 (td, *J* = 9.6, 3.4 Hz, 1H), 2.61–1.99 (m, 4H), 1.78–1.56 (m, 2H), 1.55–1.37 (m, 9H). ^13^C NMR (125 MHz, CDCl_3_) δ: 172.7, 172.1, 170.8, 170.1, 155.2, 136.4, 133.8, 132.9, 131.9, 125.5, 124.3, 124.1, 80.9, 77.3, 72.7, 72.2, 70.7, 70.4, 70.3, 68.6, 68.1, 65.7, 60.5, 53.0, 52.8, 52.6, 52.4, 52.3, 36.4, 35.8, 32.3, 31.7, 28.6, 28.3, 28.1, 28.0, 27.9, 22.7. HRMS (ESI) *m*/*z*: [M + Na]^+^ calcd. for C_20_H_32_N_2_O_7_Na, 435.2107; found, 435.2117.

*Boc-l-Hyp^OX^-l-Tyr^OX^-OMe* (**4′**; X = *n*-but-2-enyl tether): compound **4′** (5.4 mg, 23%) was obtained from compound **4** (24.4 mg, 0.0500 mmol) in a similar manner to that described for the synthesis of **1′**. White solid. Eluent for column: 50% EtOAc/*n*-hexane. *R*_f_ = 0.61 (EtOAc). ^1^H NMR (500 MHz, CDCl_3_) δ: 7.17 (d, *J* = 8.5 Hz, 1H), 6.96–6.82 (m, 2H), 6.76 (s, 1H), 6.25 (s, 1H), 5.58 (dt, *J* = 15.1, 4.9 Hz, 1H), 5.48 (dt, *J* = 15.1, 6.6, 5.6 Hz, 1H), 4.94 (ddd, *J* = 10.9, 8.9, 4.5 Hz, 1H), 4.63 (d, *J* = 5.1 Hz, 2H), 4.18–3.82 (m, 3H), 3.79 (s, 3H), 3.77–3.54 (m, 2H), 3.37 (dd, *J* = 14.1, 4.5 Hz, 1H), 3.09 (s, 1H), 2.67 (t, *J* = 12.4 Hz, 1H), 2.39–1.82 (m, 2H), 1.46 (s, 9H). HRMS (ESI) *m*/*z*: [M + Na]^+^ calcd. for C_24_H_32_N_2_O_7_Na, 483.2107; found, 483.2105.

*Boc-l-Hyp^OX^-d-Ser^OX^-OMe* (**5′**; X = *n*-but-2-enyl tether): compound **5′** (4.4 mg, 21%) was obtained from compound **2** (20.6 mg, 0.0500 mmol) in a similar manner to that described for the synthesis of **1′**. Colorless oil. Eluent for column: 70% EtOAc/*n*-hexane. *R*_f_ = 0.39 (EtOAc). ^1^H NMR (500 MHz, CDCl_3_) δ: 7.41 (br s, 0.6H), 7.17 (br s, 0.4H), 5.95–5.85 (m, 1H), 5.85–5.66 (m, 1H), 4.55–4.18 (m, 4H), 4.14 (t, *J* = 4.1 Hz, 1H), 4.02–3.94 (m, 1H), 3.93–3.84 (m, 2H), 3.82 (m, 1.2H), 3.77 (s, 1.8H), 3.73–3.56 (m, 2H), 3.45 (dd, *J* = 12.2, 4.1 Hz, 1H), 2.62 (d, *J* = 15.2 Hz, 0.6H), 2.50 (d, *J* = 14.0 Hz, 0.4H), 2.29–2.12 (m, 1H), 1.45 (d, *J* = 8.9 Hz, 9H). HRMS (ESI) *m*/*z*: [M + Na]^+^ calcd. for C_18_H_28_N_2_O_7_Na, 407.1794; found, 407.1790.

*Boc-l-Hyp^OX^-l-Thr^OX^-OMe* (**6′**; X = *n*-but-2-enyl tether): compound **6′** (7.8 mg, 39%) was obtained from compound **6** (21.3 mg, 0.0500 mmol) in a similar manner to that described for the synthesis of **1′**. Colorless oil. Eluent for column: 70% EtOAc/*n*-hexane. *R*_f_ = 0.32 (EtOAc). ^1^H NMR (500 MHz, CDCl_3_) δ: 7.02 (d, *J* = 8.2 Hz, 1H), 5.86 (dt, *J* = 11.5, 6.7 Hz, 1H), 5.78 (dt, *J* = 11.5, 6.2 Hz, 1H), 4.72–4.54 (m, 1H), 4.39–4.21 (m, 2H), 4.16 (dd, *J* = 11.8, 6.5 Hz, 1H), 4.12–4.06 (m, 1H), 4.01 (dd, *J* = 11.8, 6.2 Hz, 1H), 3.89–3.76 (m, 2H), 3.72 (s, 3H), 3.76–3.62 (m, 1H), 3.45 (dd, *J* = 12.0, 3.3 Hz, 1H), 2.51 (d, *J* = 14.8 Hz, 1H), 2.27–2.14 (m, 1H), 1.43 (s, 9H), 1.21 (d, *J* = 6.3 Hz, 3H). HRMS (ESI) *m*/*z*: [M + Na]^+^ calcd. for C_19_H_30_N_2_O_7_Na, 421.1951; found, 421.1958.

*Boc-l-Hyp^OX^-(S)-Ala(Et^X^)-OMe* (**7′**; X = *n*-but-2-enyl tether): compound **7′** (8.5 mg, 43%) was obtained from compound **7** (21.2 mg, 0.0500 mmol) in a similar manner to that described for the synthesis of **1′**. Colorless oil. Eluent for column: 5% MeOH in CHCl_3_. *R*_f_ = 0.52 (10% MeOH in CHCl_3_). ^1^H NMR (500 MHz, CDCl_3_) δ: 7.52 (s, 1H), 5.90–5.77 (m, 1H), 5.65 (dt, *J* = 10.5, 7.3 Hz, 1H), 4.42–4.22 (m, 1H), 4.15–3.92 (m, 2H), 3.74 (s, 3H), 3.70–3.43 (m, 3H), 2.83–2.64 (m, 1H), 2.62–2.48 (m, 1H), 2.22–1.86 (m, 4H), 1.64 (s, 3H), 1.52–1.43 (m, 9H), 1.51–1.43 (m, 1H), 1.22–1.12 (m, 1H). HRMS (ESI) *m*/*z*: [M + Na]^+^ calcd. for C_20_H_32_N_2_O_6_Na, 419.2158; found, 419.2166.

### 3.4. Synthesis of Stapled Octapeptides ***9*** and ***10***

*Boc-l-Hyp^OX^-l-Ser^OX^-[(l-Leu)_2_-Ac_5_c]_2_-OMe* (**9**; X = *n*-but-2-enyl tether): compound **9** (10.2 mg, 52%) was obtained from compound **8** [19] (20.0 mg, 0.0184 mmol) in a similar manner to that described for the synthesis of **1′**. Eluent for column: 80% EtOAc/*n*-hexane. White amorphous. *R*_f_ = 0.26 (EtOAc). ^1^H NMR (500 MHz, CDCl_3_) δ: 7.73 (d, *J* = 2.3 Hz, 1H), 7.43 (d, *J* = 8.0 Hz, 1H), 7.37 (d, *J* = 4.8 Hz, 1H), 7.27–7.24 (m, 1H), 7.24–7.18 (m, 3H), 6.08 (dd, *J* = 10.9, 6.6 Hz, 1H), 6.04 (dd, *J* = 10.9, 5.8 Hz, 1H), 4.46 (ddd, *J* = 11.7, 5.3, 2.3 Hz, 1H), 4.39–4.30 (m, 2H), 4.26–4.15 (m, 5H), 4.01 (dd, *J* = 11.4, 5.3 Hz, 1H), 3.96–3.88 (m, 2H), 3.74 (dd, *J* = 10.0, 5.3 Hz, 1H), 3.71–3.68 (m, 1H), 3.67 (s, 3H), 3.66–3.60 (m, 1H), 3.46 (dd, *J* = 12.1, 3.4 Hz, 1H), 2.66 (dt, *J* = 13.5, 8.1 Hz, 1H), 2.45–2.30 (m, 2H), 2.26 (ddd, *J* = 13.6, 8.5, 6.7 Hz, 1H), 2.22–2.10 (m, 3H), 2.10–2.02 (m, 1H), 1.97–1.55 (m, 28H), 1.49 (s, 9H), 1.00–0.83 (m, 24H). HRMS (ESI) *m*/*z*: [M + Na]^+^ calcd. for C_54_H_90_N_8_O_13_Na, 1081.6525; found, 1081.6536.

*Boc-l-Hyp^OX^-l-Ser^OX^-[(l-Leu)_2_-Ac_5_c]_2_-OMe* (**10**; X = *n*-butyl tether): to a solution of peptide **9** (10.2 mg, 0.00963 mmol) in MeOH (2 mL) was added 10% Pd/C (10 mg) at room temperature and the reaction mixture was stirred at room temperature overnight. The resultant dark suspension was filtered through a short plug of celite (MeOH), and the organics were concentrated under vacuum. The crude material was purified by preparative TLC (EtOAc) to give **10** (8.5 mg, 83%) as white amorphous. *R*_f_ = 0.31 (EtOAc). ^1^H NMR (500 MHz, CDCl_3_) δ: 7.70 (s, 1H), 7.46 (d, *J* = 5.0 Hz, 1H), 7.44 (d, *J* = 8.0 Hz, 1H), 7.26–7.24 (m, 2H), 7.23 (d, *J* = 5.6 Hz, 2H), 4.35 (ddd, *J* = 11.4, 8.1, 3.0 Hz, 1H), 4.28 (ddd, *J* = 11.0, 4.8, 1.5 Hz, 1H), 4.24–4.16 (m, 2H), 4.14 (d, *J* = 10.9 Hz, 1H), 4.04 (t, *J* = 3.5 Hz, 1H), 3.98 (dd, *J* = 11.1, 5.1 Hz, 1H), 3.93 (dt, *J* = 9.6, 4.5 Hz, 1H), 3.83 (dd, *J* = 11.9, 2.2 Hz, 1H), 3.70 (dd, *J* = 9.3, 1.6 Hz, 1H), 3.67 (s, 3H), 3.63 (dd, *J* = 9.3, 2.7 Hz, 1H), 3.58 (dt, *J* = 9.3, 3.2 Hz, 1H), 3.54 (t, *J* = 11.2 Hz, 1H), 3.44–3.36 (m, 2H), 2.65 (dt, *J* = 13.6, 8.3 Hz, 1H), 2.38 (ddd, *J* = 15.1, 11.1, 4.3 Hz, 1H), 2.27 (dd, *J* = 13.8, 7.5 Hz, 1H), 2.24–2.03 (m, 5H), 1.96–1.66 (m, 22H), 1.66–1.57 (m, 4H), 1.52 (s, 9H), 0.99–0.93 (m, 9H), 0.92–0.85 (m, 15H). X-ray crystallographic data and CIF file of compound **10** are provided in the Supplementary Materials.

## Figures and Tables

**Table 1:**
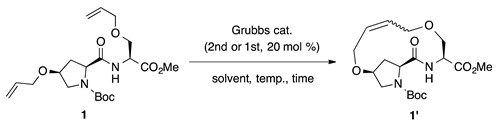
Screening of reaction conditions for ring-closing metathesis of dipeptide **1**.

Entry ^1^	Catalyst (mol %)	Solvent (mM)	Temp. (°C)	Time (h)	Yield (%)	*E*/*Z* Ratio ^2^
1	Grubbs 2nd (20)	CH_2_Cl_2_ (20)	rt	2	55	1.0:5.6
2	Grubbs 1st (20)	CH_2_Cl_2_ (20)	rt	2	53	1.0:4.8
3	Grubbs 2nd (20)	toluene (20)	rt	2	37	1.0:3.0
4	Grubbs 2nd (20)	DCE (20)	rt	2	39	1.0:4.8
5	Grubbs 2nd (20)	THF (20)	rt	2	27	1.0:4.4
6	Grubbs 2nd (20)	CH_2_Cl_2_ (5)	rt	2	76	1.0:5.0
7	Grubbs 1st (20)	CH_2_Cl_2_ (5)	rt	2	69	1.0:4.7
8	Grubbs 2nd (20)	CH_2_Cl_2_ (5)	reflux	2	28	1.0:4.9
9	Grubbs 2nd (20)	CH_2_Cl_2_ (5)	reflux	0.5	28	1.0:5.3

^1^ Condition: 0.05 mmol of **1**. ^2^ Determined by ^1^H NMR.

**Table 2:** Crystal and diffraction parameters of peptide **10**.

Empirical Formula	C_54_ H_92_ N_8_ O_13_, C_3_ H_7_ N O
Formula weight	1134.45
Crystal dimensions (mm)	0.403 × 0.275 × 0.250
Data collection temp. (K)	93
Crystal system	orthorhombic
Lattice parameters	
*a*, *b*, *c* (Å)	11.357, 19.104, 29.332
*α*, *β*, *γ* (°)	90, 90, 90
*V* (Å^3^)	6363.84
Space group	*P* 2_1_ 2_1_ 2_1_
*Z* value	4
*D* calc (g/cm^3^)	1.184
*μ* (MoKα) (cm^−1^)	0.692
No. of variable	765
No. of observations	11273 (*I* > 2δ(I))
R_1_ (*I* > 2δ(I))	0.0275
*w*R_2_	0.0707
Crystallizing solvent	DMF/H_2_O

**Table 3:** Intra and intermolecular H-bond parameters for peptide **10**.

Donor D–H	Acceptor A	Distance [Å] D···A	Angle [°] D–H···A	Symmetry Operations
N_4_-H	O_0_	3.09	163.6	*x*,*y*,*z*
N_5_-H	O_1_	2.98	168.6	*x*,*y*,*z*
N_6_-H	O_2_	2.91	157.2	*x*,*y*,*z*
N_7_-H	O_3_	3.14	139.7	*x*,*y*,*z*
N_7_-H	O_4_	3.37 ^1^	136.7	*x*,*y*,*z*
N_8_-H	O_4_	3.23	102.9 ^2^	*x*,*y*,*z*
N_8_-H	O_5_	3.40 ^1^	162.7	*x*,*y*,*z*
N_2_-H	O_Hyp4_	2.93	137.6	*x*,*y*,*z*
N_3_-H	O_DMF_	2.90	159.8	1/2–*x*,1–*y*,–1/2 + *z*

^1^ Distance is a little long for an intramolecular hydrogen bond. ^2^ N–H···O angle is too small for a hydrogen bond.

## Data Availability

The data presented in this study are available on request from the corresponding author.

## Supplementary Materials

The following are available online at https://www.mdpi.com/article/10.3390/ijms22105364/s1: ^1^H and ^13^C NMR spectra of compounds **2**–**7**, **1′**–**7′**, **9**, and **10**; X-ray crystallographic data of compound **10**, and CIF file of compound **10**.
