# Supplementary material for: X-ray Crystallographic Structure of α-Helical Peptide Stabilized by Hydrocarbon Stapling at i,i + 1 Positions
Source: Int J Mol Sci. 2021 May 19;22(10):5364. doi: 10.3390/ijms22105364 (PMC8160927; doi:10.3390/ijms22105364)

Supplementary Materials

X-ray Crystallographic Structure of α-Helical Peptide Stabilized by Hydrocarbon Stapling at *i,i*+1 Positions

Yui Makura [1], Atsushi Ueda [1,*], Takuma Kato [2], Akihiro Iyoshi [1], Mei Higuchi [1], Mitsunobu Doi [2] and Masakazu Tanaka [1,*]

[1] Graduate School of Biomedical Sciences, Nagasaki University, 1-14 Bunkyo-machi, Nagasaki 852-8521, Japan

[2] Faculty of Pharmacy, Osaka Medical and Pharmaceutical University, Osaka 569-1094, Japan

E-mail for A.U.: aueda@nagasaki-u.ac.jp

E-mail for M.T.: matanaka@nagasaki-u.ac.jp

Table of Contents

1. X-Ray crystallographic data of stapled peptide **10** ········· S2

2. Copies of ^{1}H and ^{13}C NMR spectra ········· S5

1. X-ray crystallographic data of stapled peptide 10

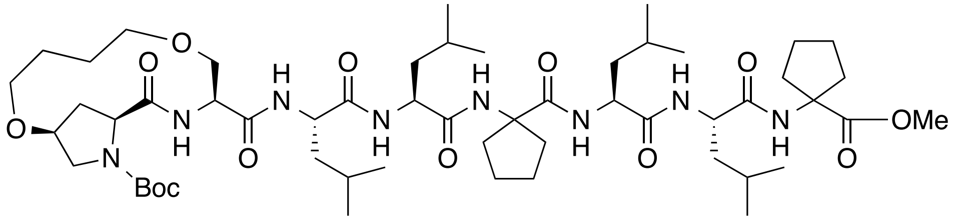

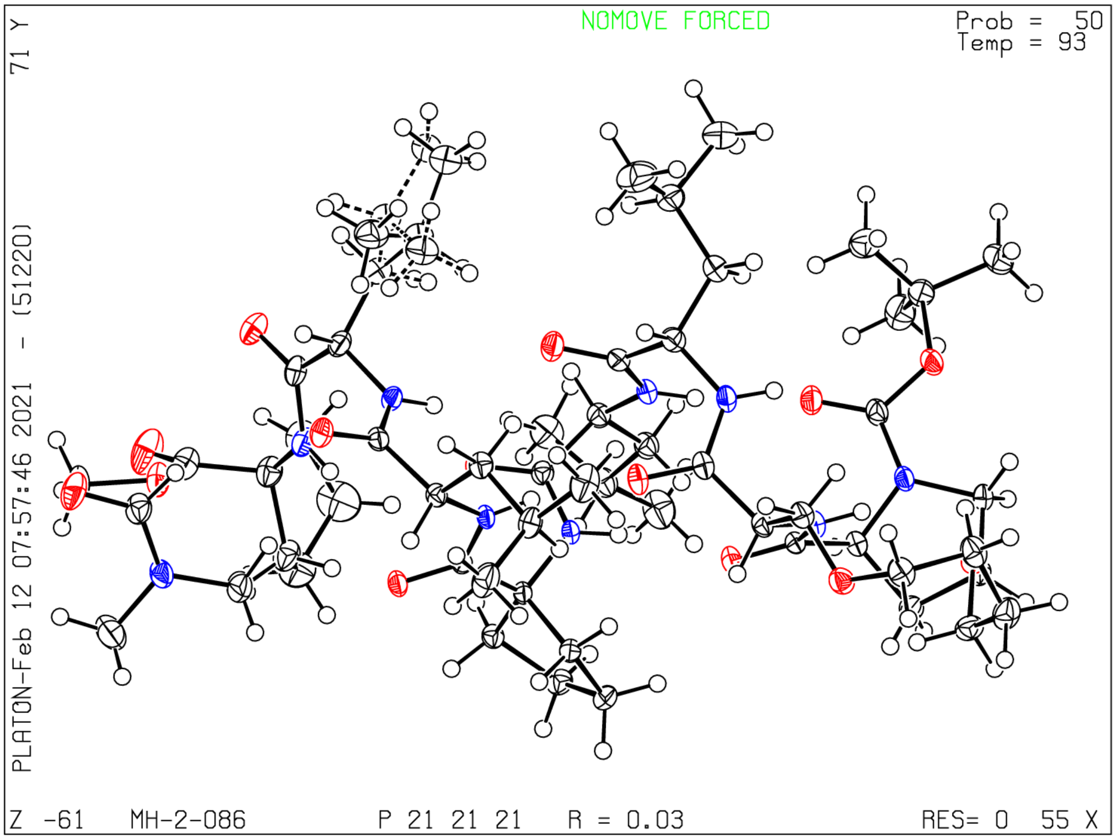

Figure S1. ORTEP drawing of stapled peptide **10** (ellipsoids at 50% probability).

Table S1. Selected torsion angles ω, ϕ, ψ, and χ [°] for stapled peptide **10**, as determined by an X-ray crystallographic analysis.

ω0	–170.4
ϕ1	–67.9
ψ1	–45.6
ω1	–177.3
ϕ2	–66.4
ψ2	–44.4
ω2	179.8
ϕ3	–61.2
ψ3	–43.0
ω3	177.3
ϕ4	–59.5
ψ4	–53.3
ω4	–176.1
ϕ5	–57.1
ψ5	–46.2
ω5	–169.4
ϕ6	–75.3
ψ6	–21.7
ω6	–179.5
ϕ7	–102.3
ψ7	7.9
ω7	–174.4
ϕ8	–60.6
ψ8	–41.6
ω8	–170.4
χ1	19.1
χ2	–70.4
χ3	–172.8
χ4	172.3
χ5	–81.9
χ5’	75.0
χ6	–65.9
χ7	–58.2
χ8	–143.3
χ8’	115.6

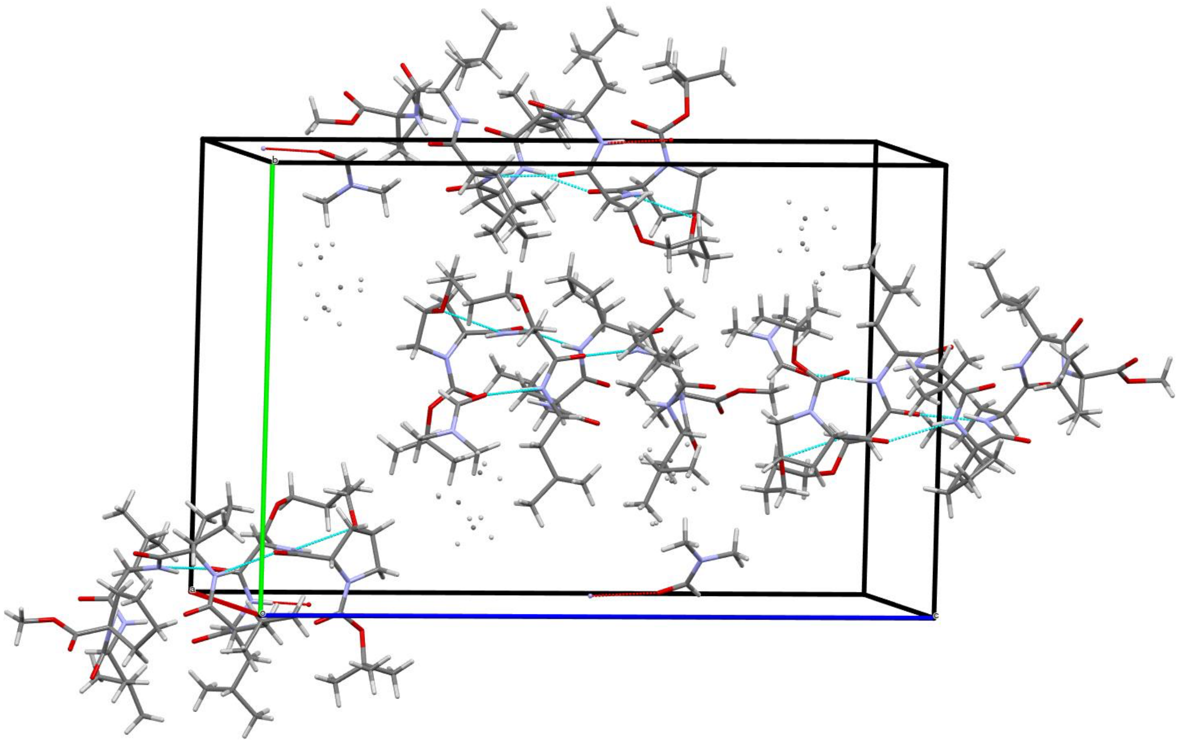

Figure S2. Packing structure of stapled peptide **10** in the crystal state.

1. NMR spectra.

^{1}H NMR of **2** (500 MHz, $CDCl_3$)

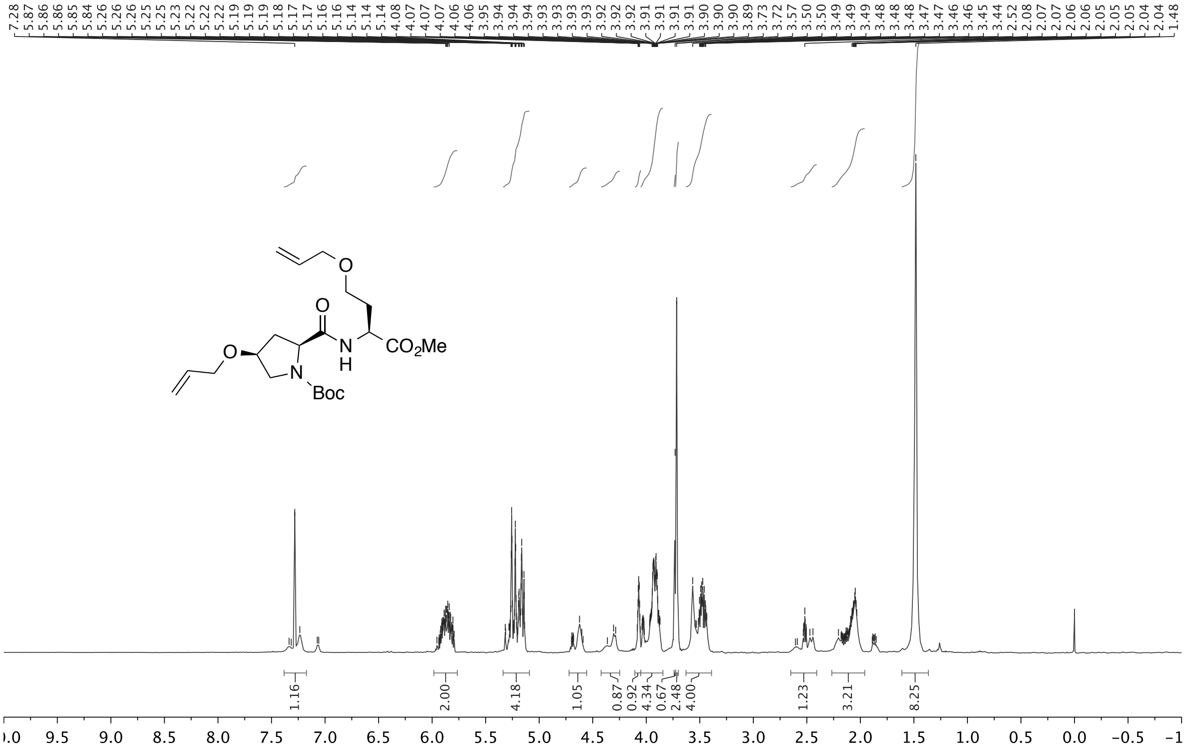

^{13}C NMR of **2** (125 MHz, $CDCl_3$)

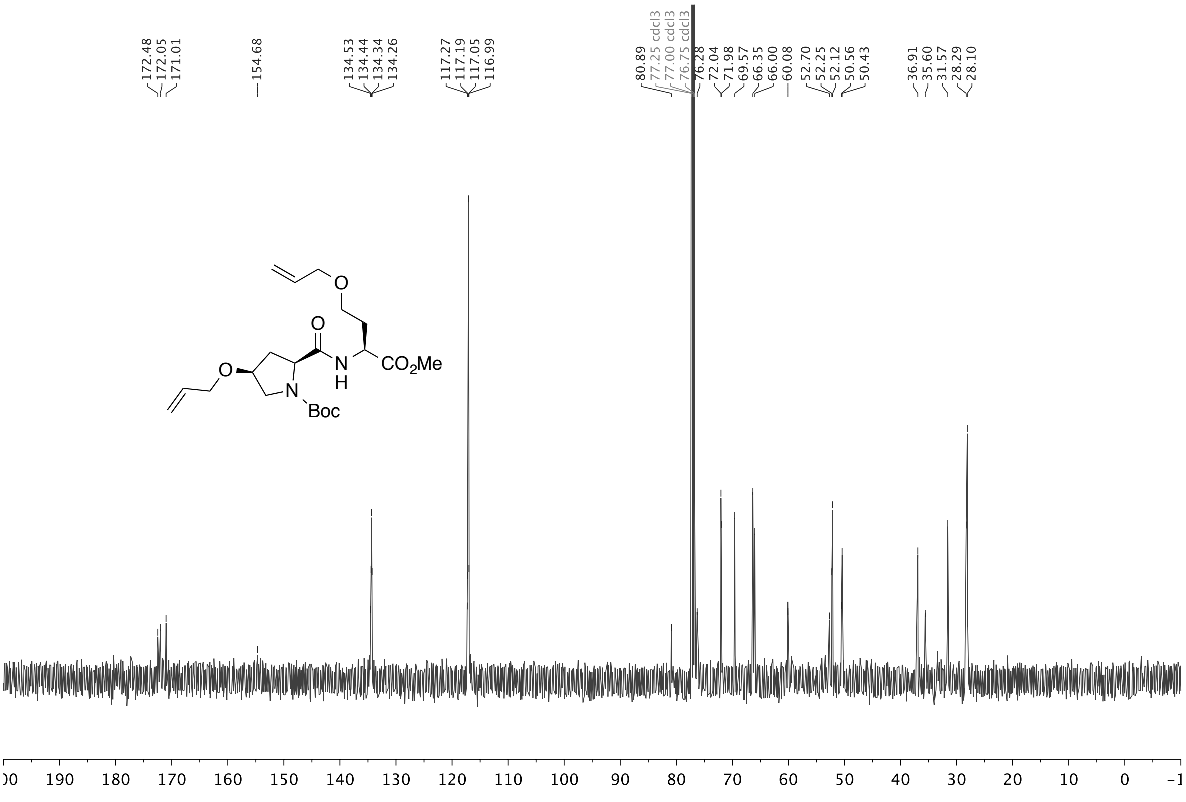

^{1}H NMR of **3** (500 MHz, $CDCl_3$)

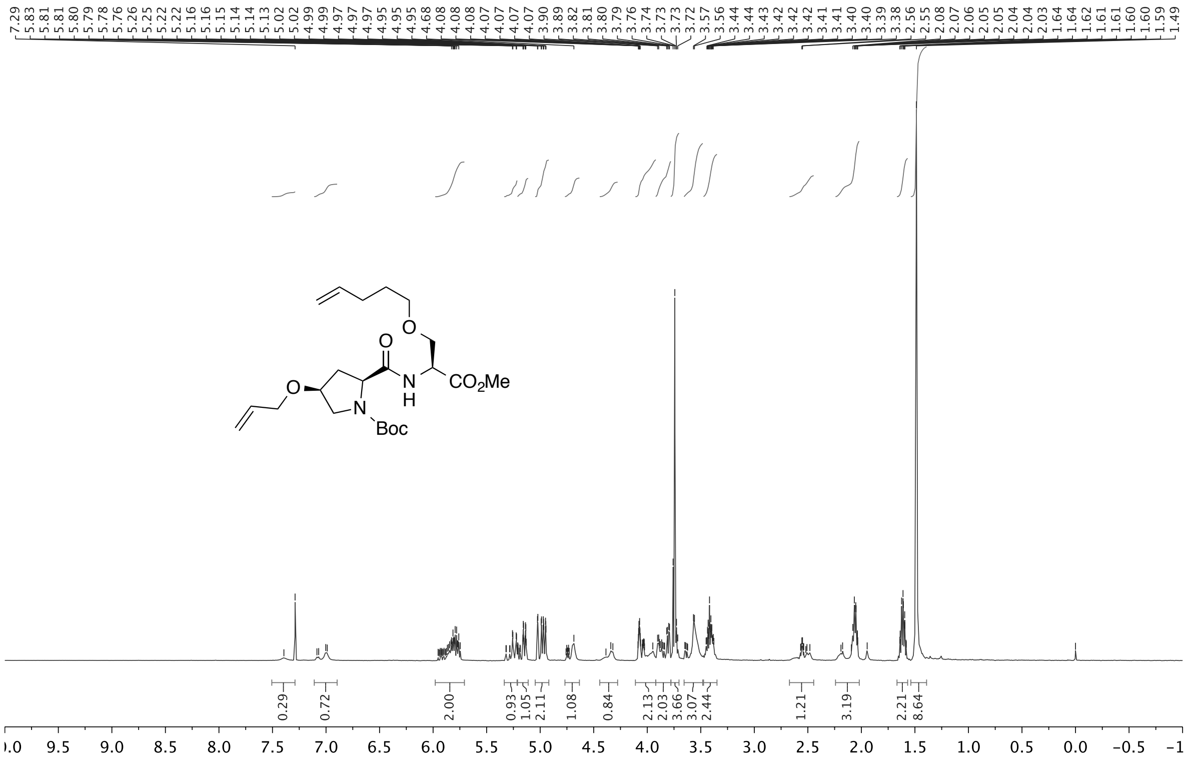

^{13}C NMR of **3** (125 MHz, $CDCl_3$)

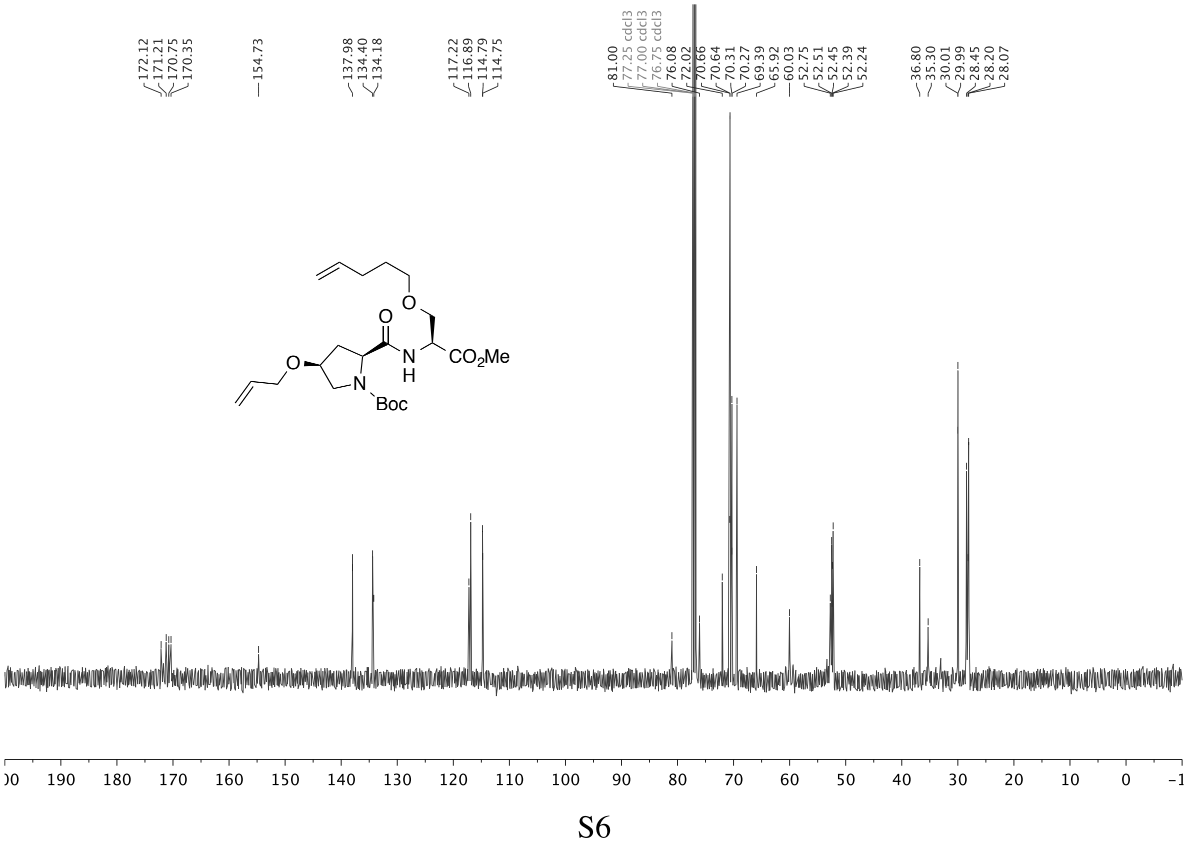

^{1}H NMR of **4** (500 MHz, $CDCl_3$)

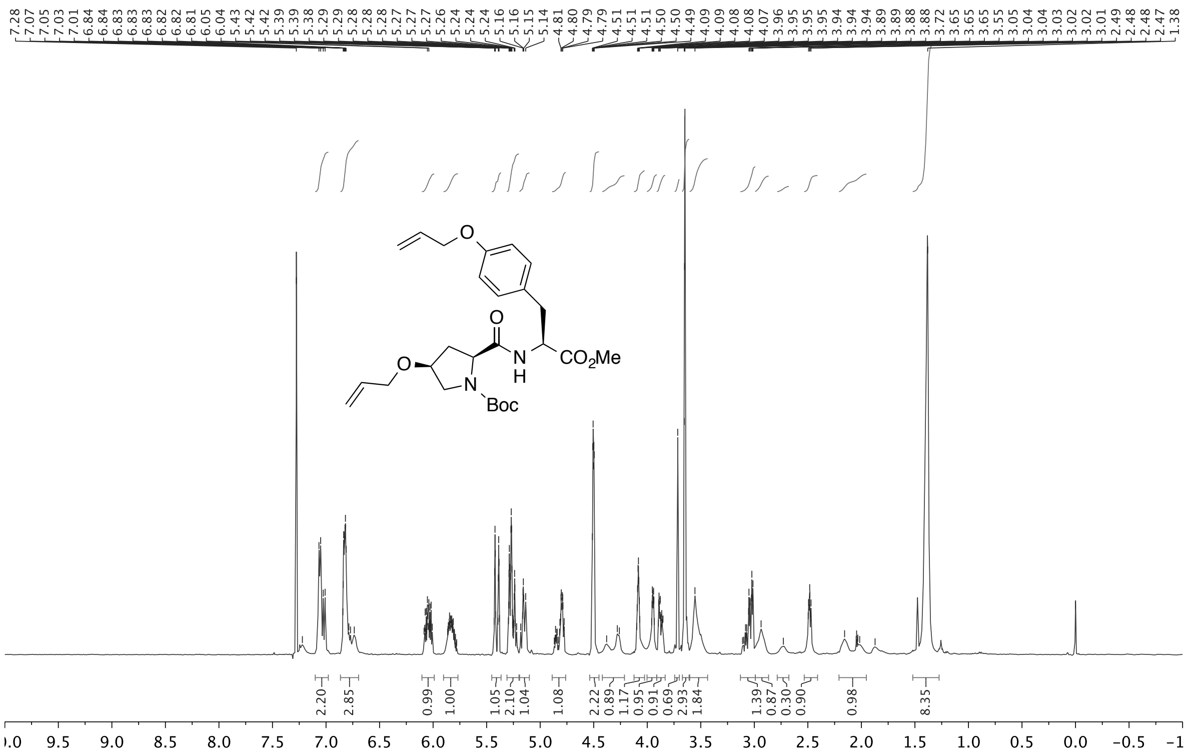

^{13}C NMR of **4** (125 MHz, $CDCl_3$)

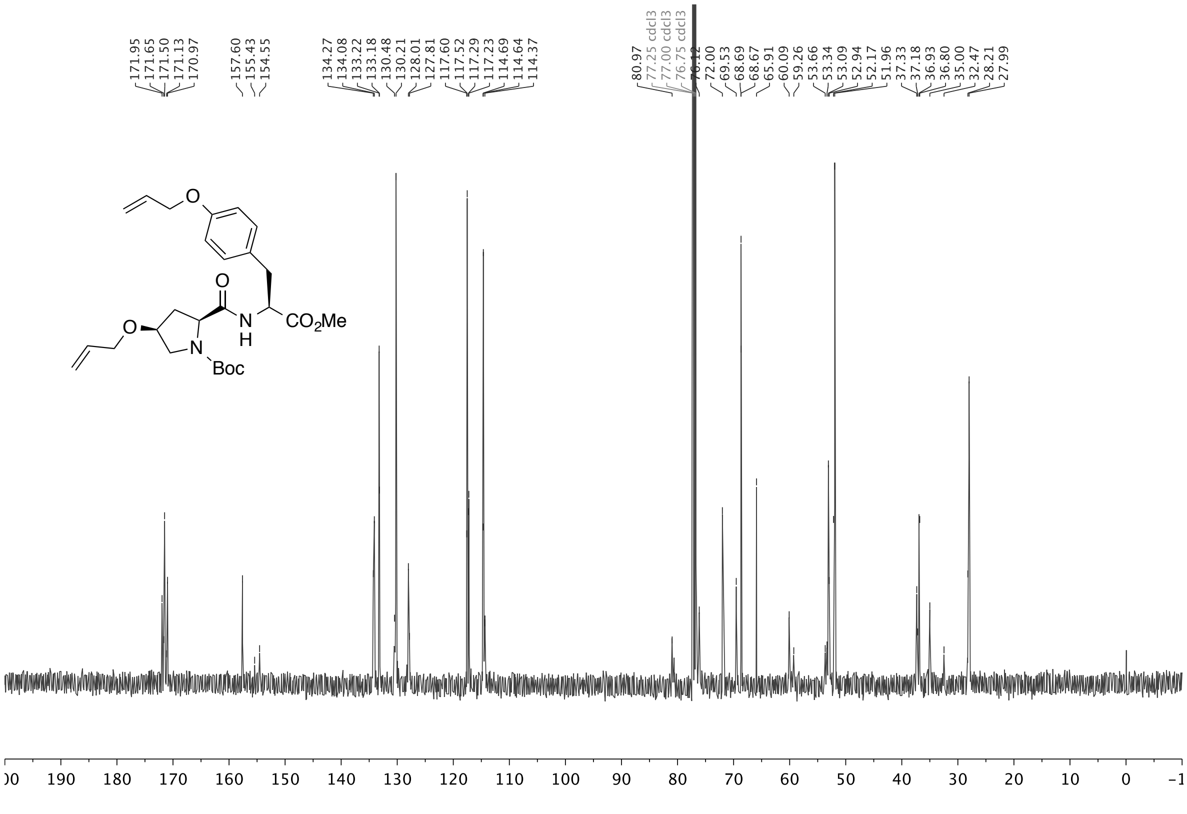

^{1}H NMR of **5** (500 MHz, $CDCl_3$)

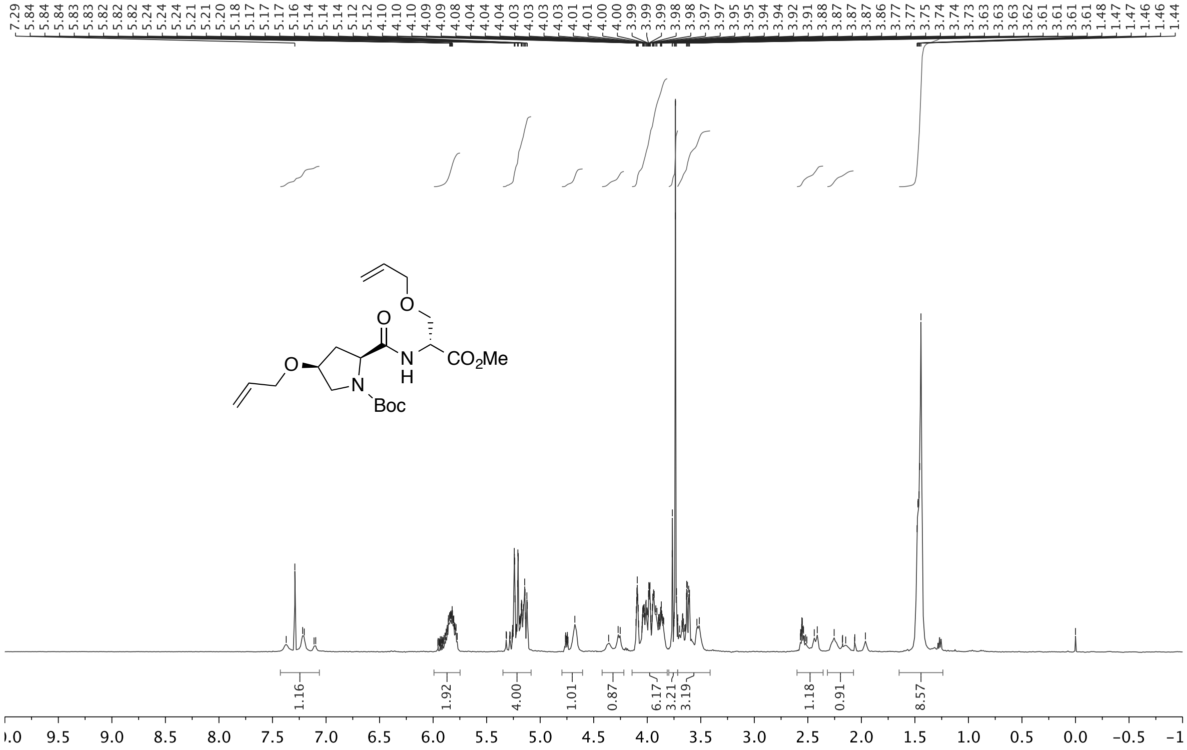

^{13}C NMR of **5** (125 MHz, $CDCl_3$)

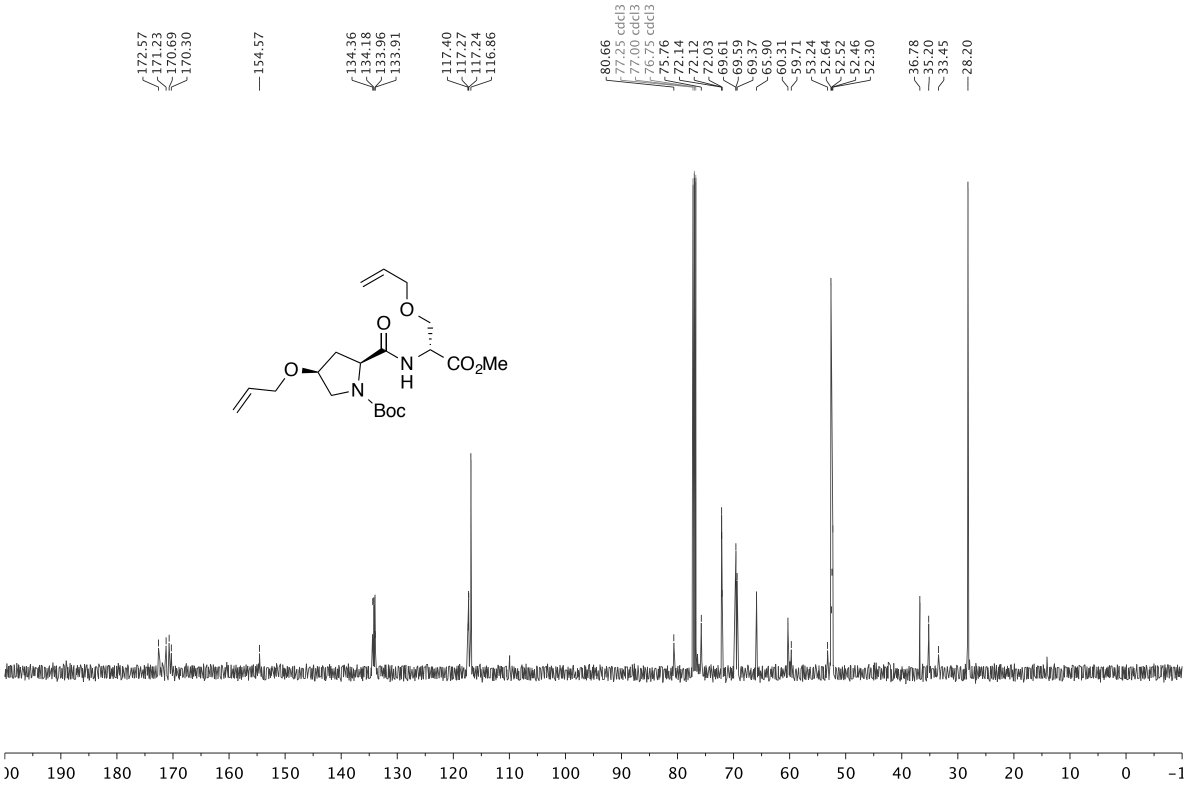

^{1}H NMR of **6** (400 MHz, $CDCl_3$)

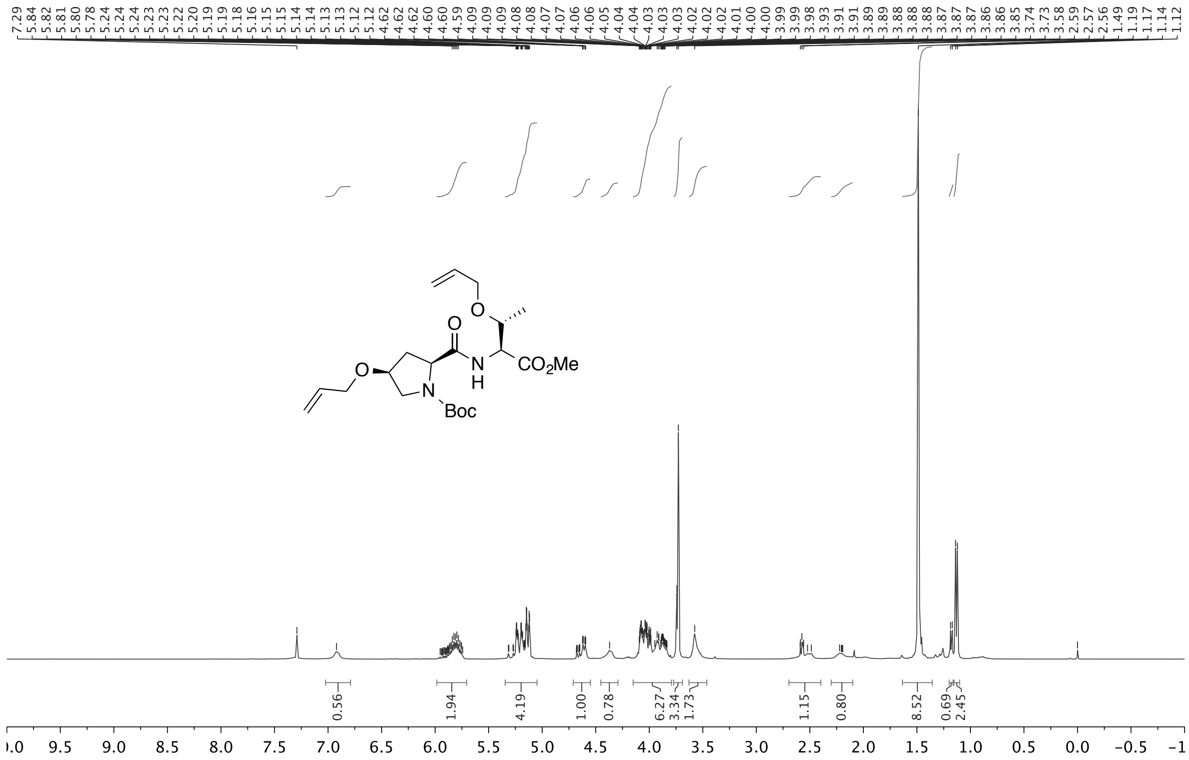

^{13}C NMR of **6** (100 MHz, $CDCl_3$)

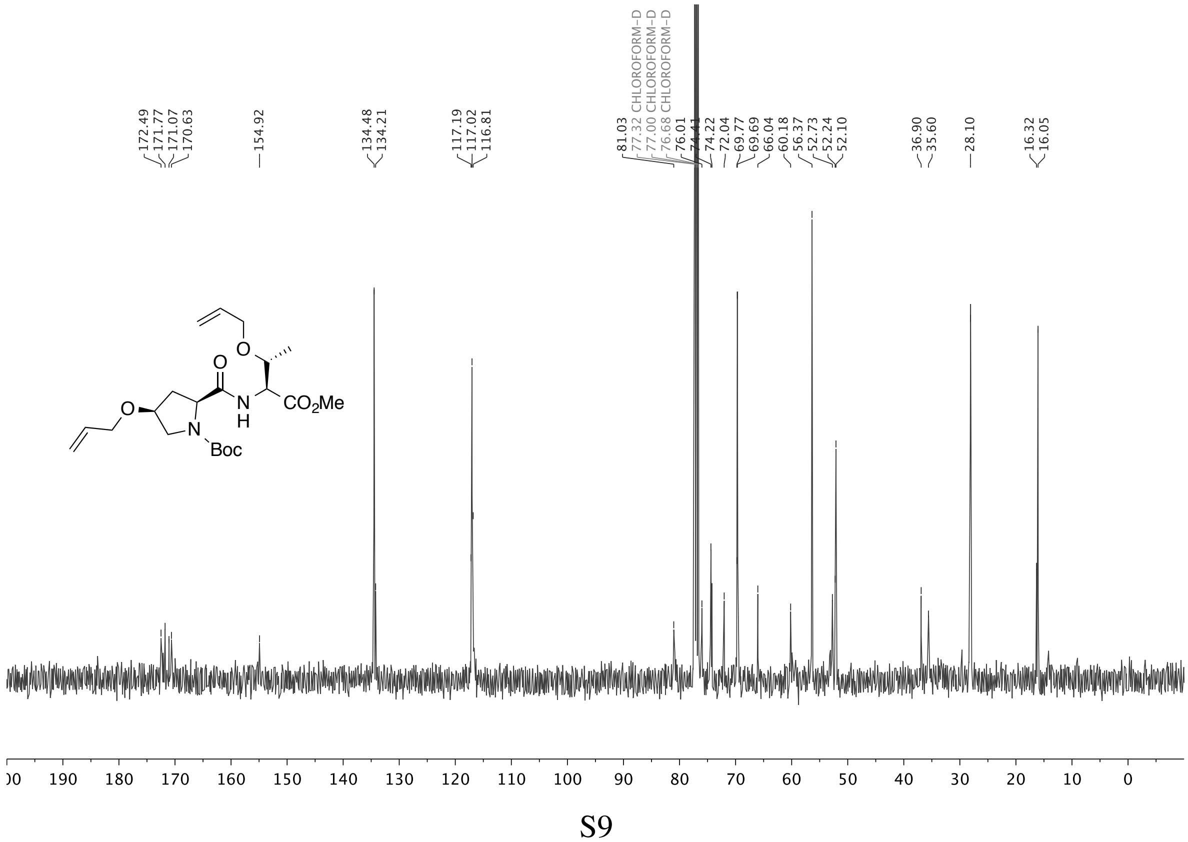

^{1}H NMR of **7** (500 MHz, $CDCl_3$)

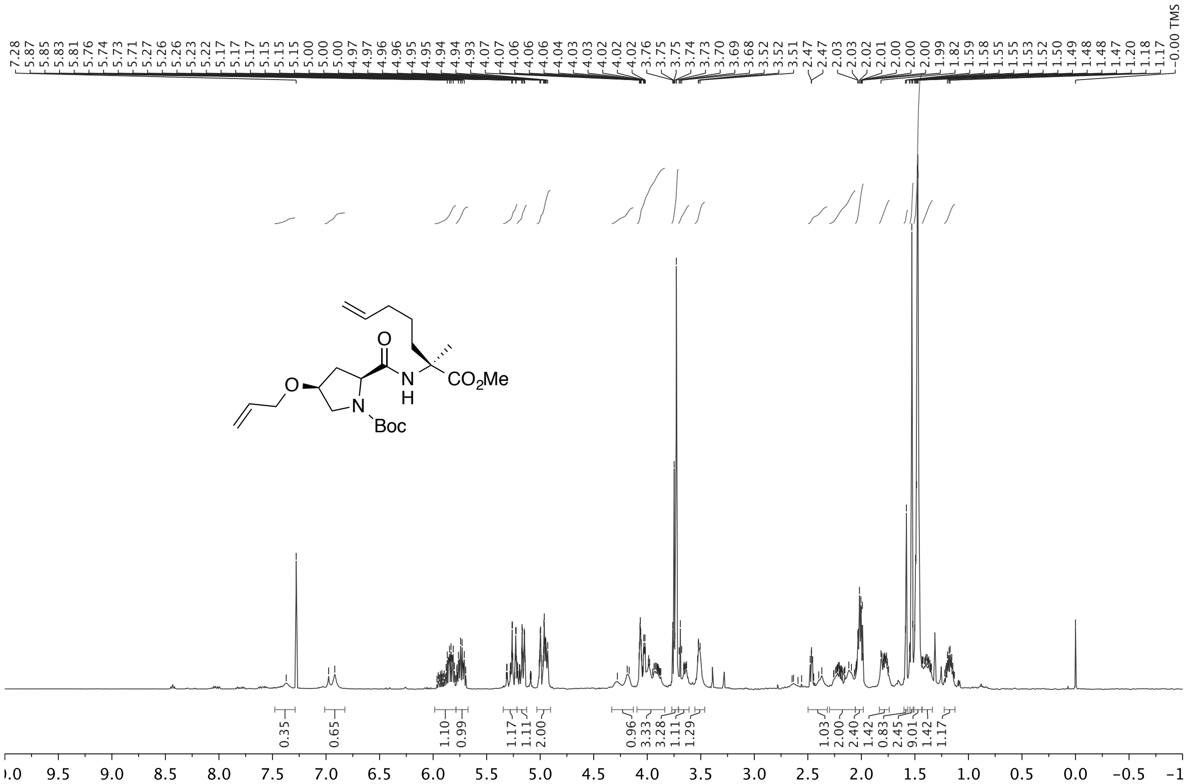

^{13}C NMR of **7** (125 MHz, $CDCl_3$)

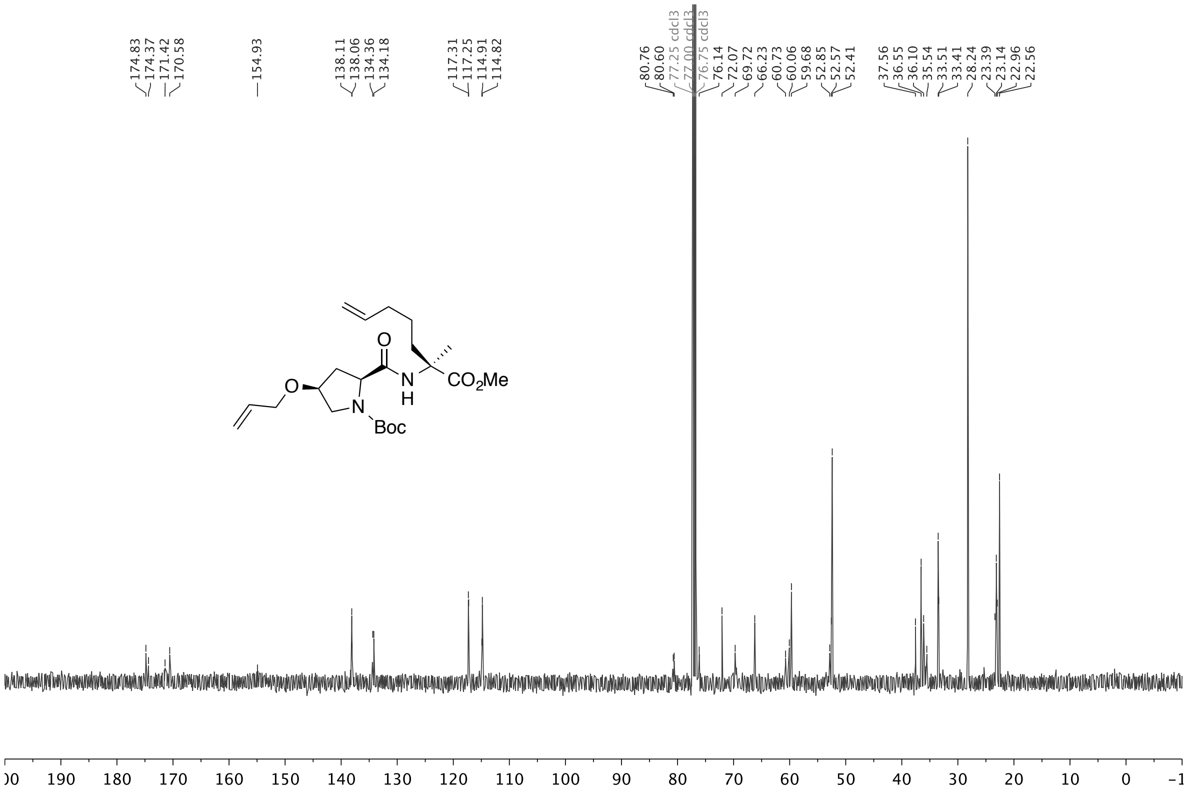

^{1}H NMR of **1'** (500 MHz, $CDCl_3$)

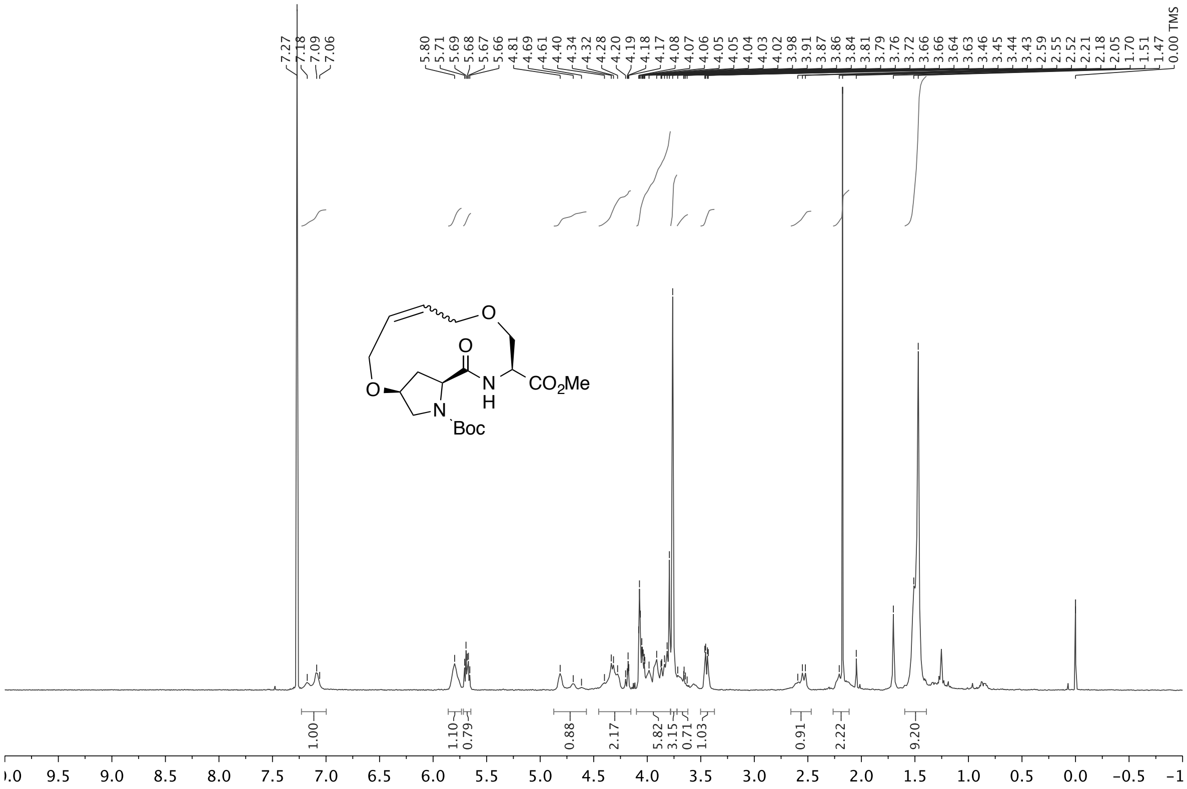

^{13}C NMR of **1'** (125 MHz, $CDCl_3$)

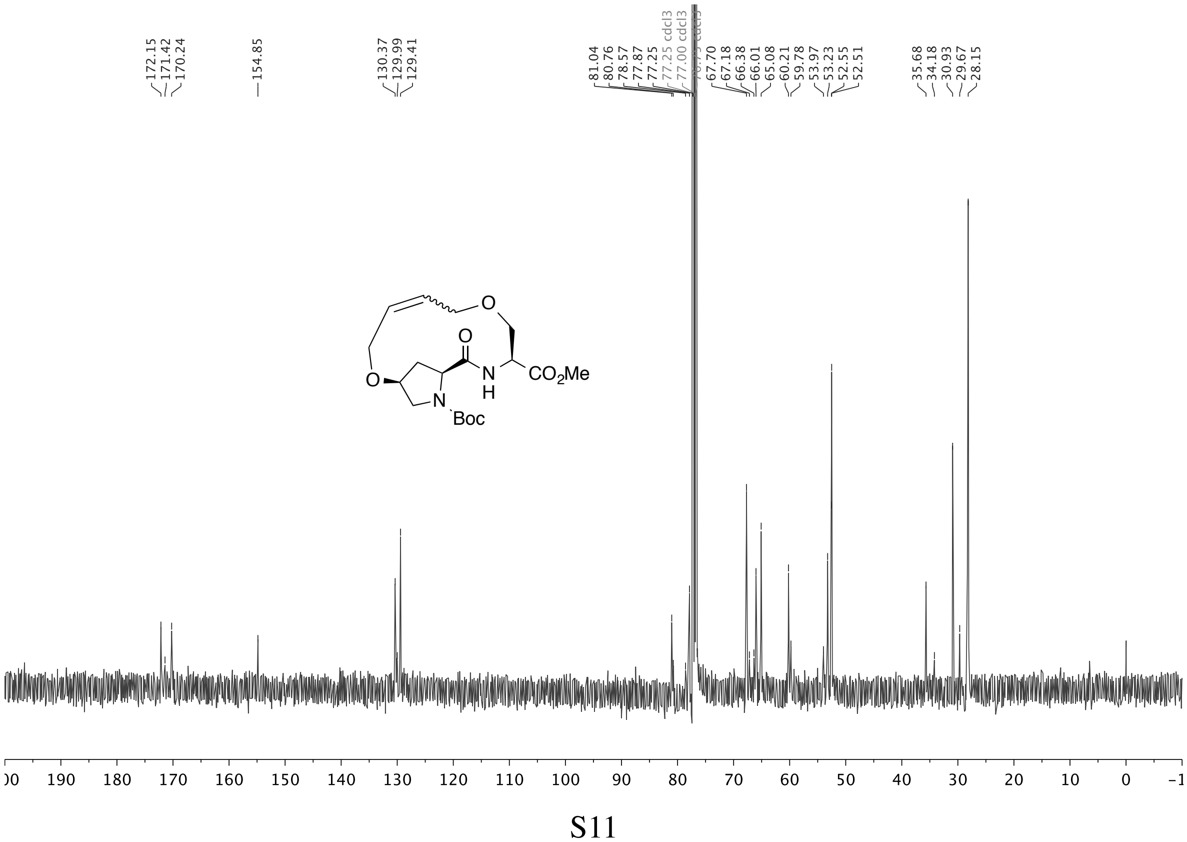

^{1}H NMR of **2’** (500 MHz, $CDCl_3$)

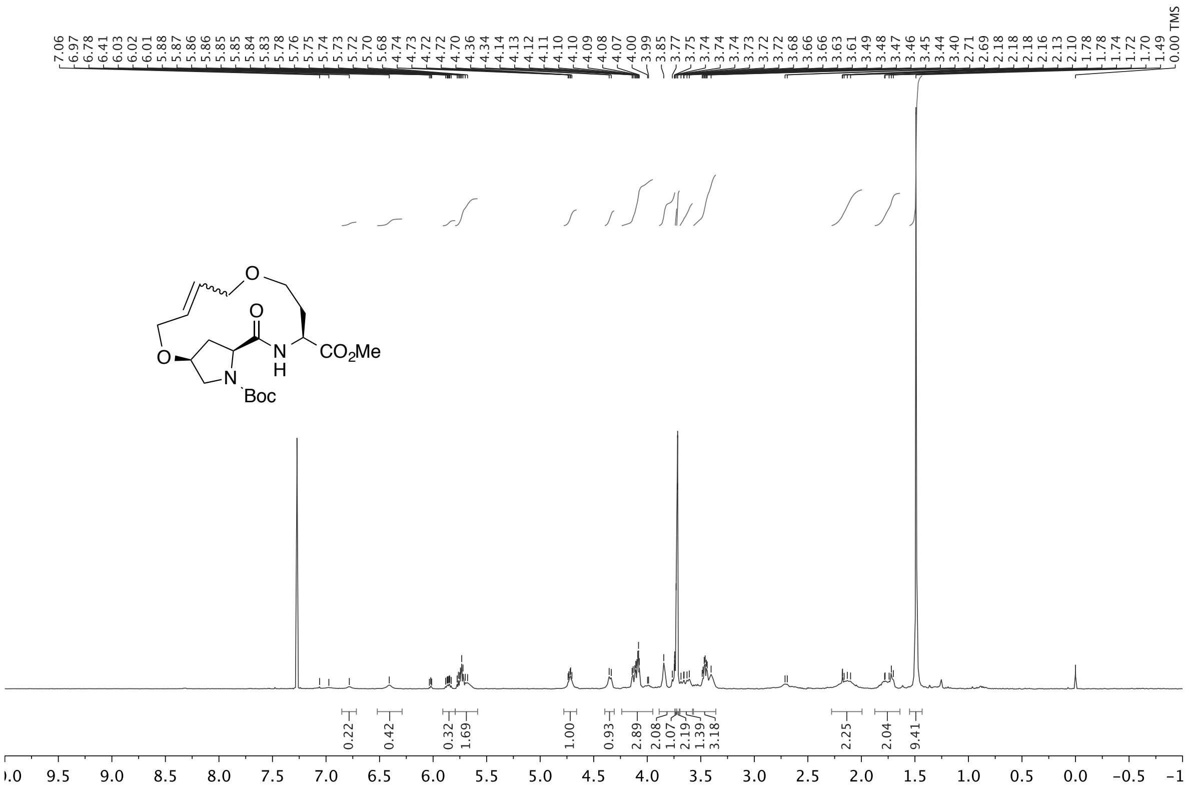

^{13}C NMR of **2’** (125 MHz, $CDCl_3$)

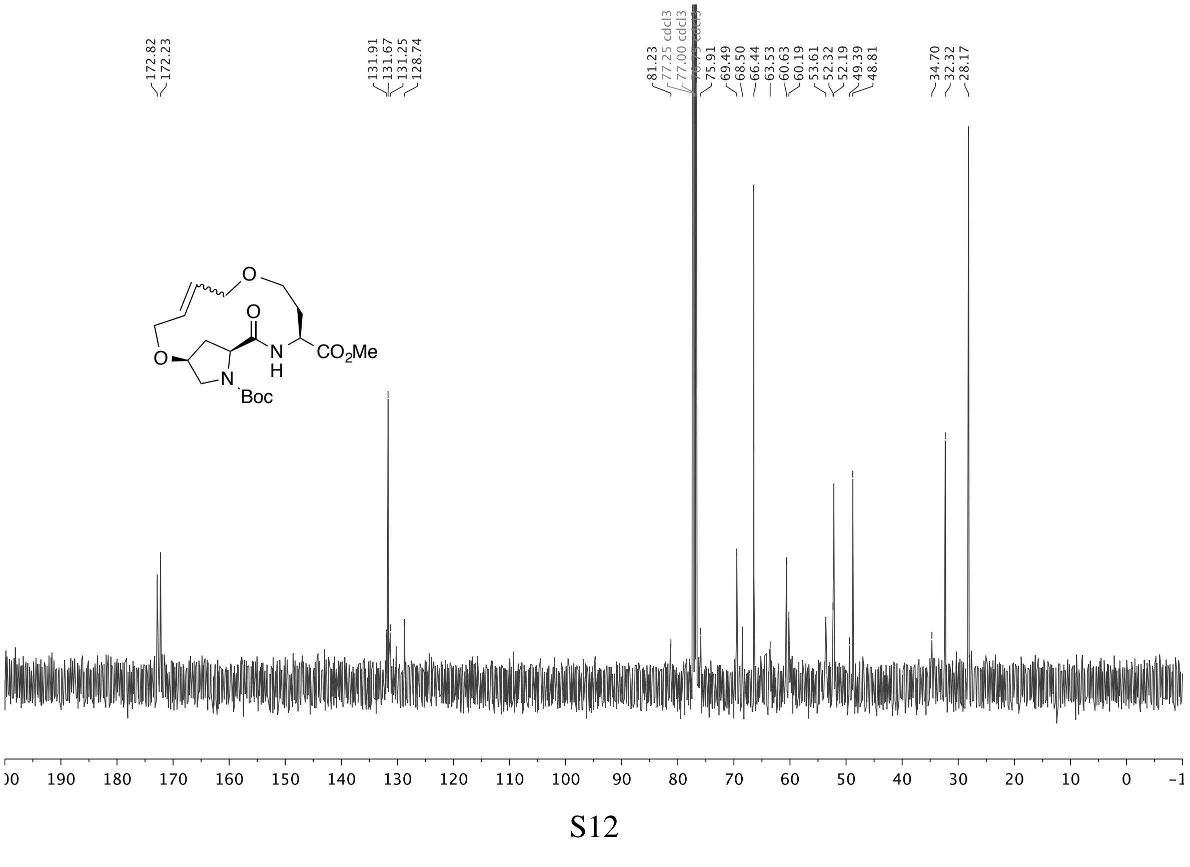

^{1}H NMR of **3'** (500 MHz, $CDCl_3$)

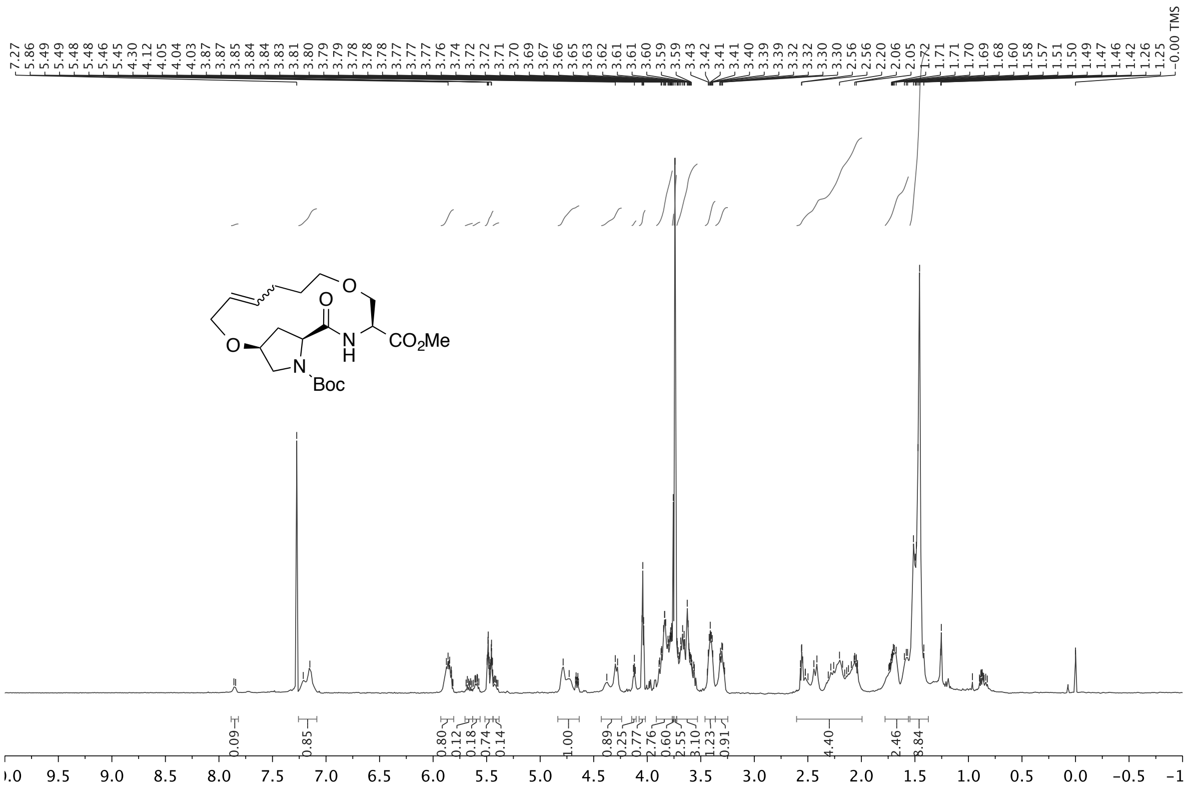

^{13}C NMR of **3'** (125 MHz, $CDCl_3$)

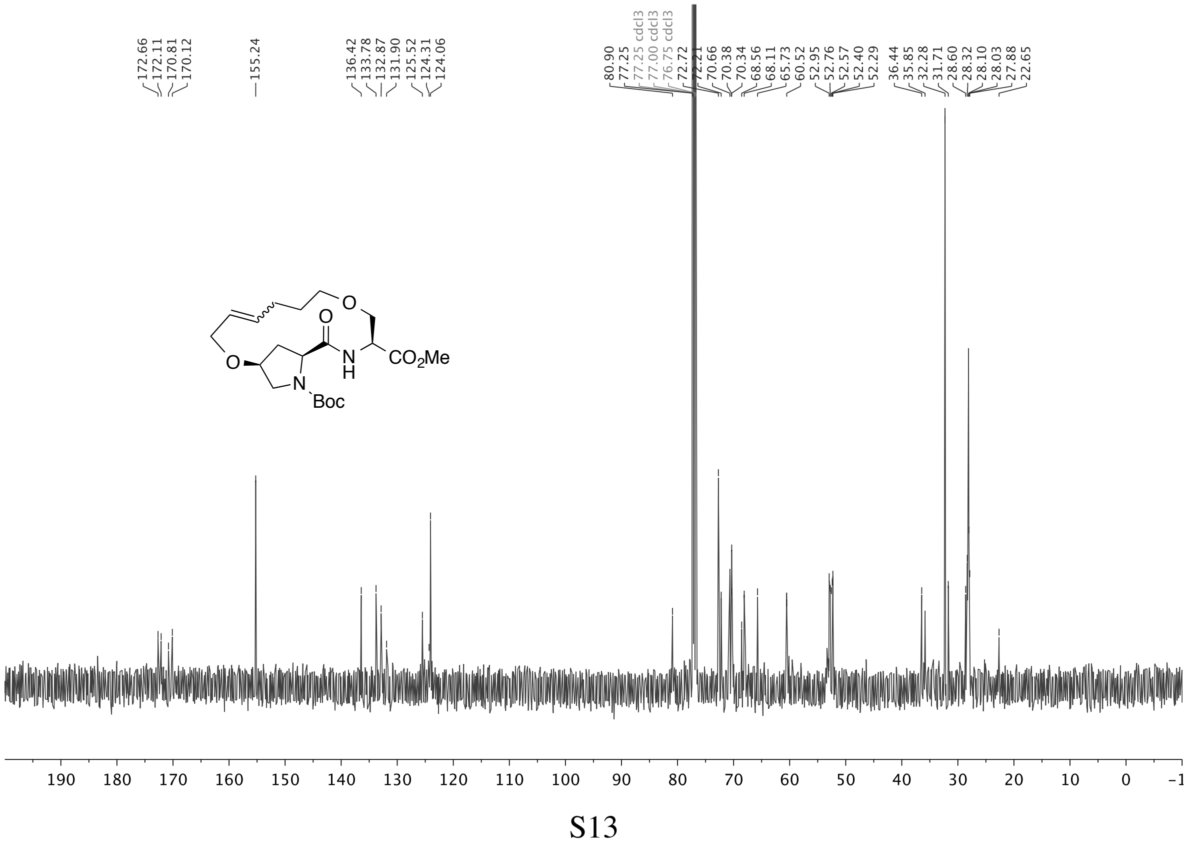

^{1}H NMR of **4’** (500 MHz, $CDCl_3$)

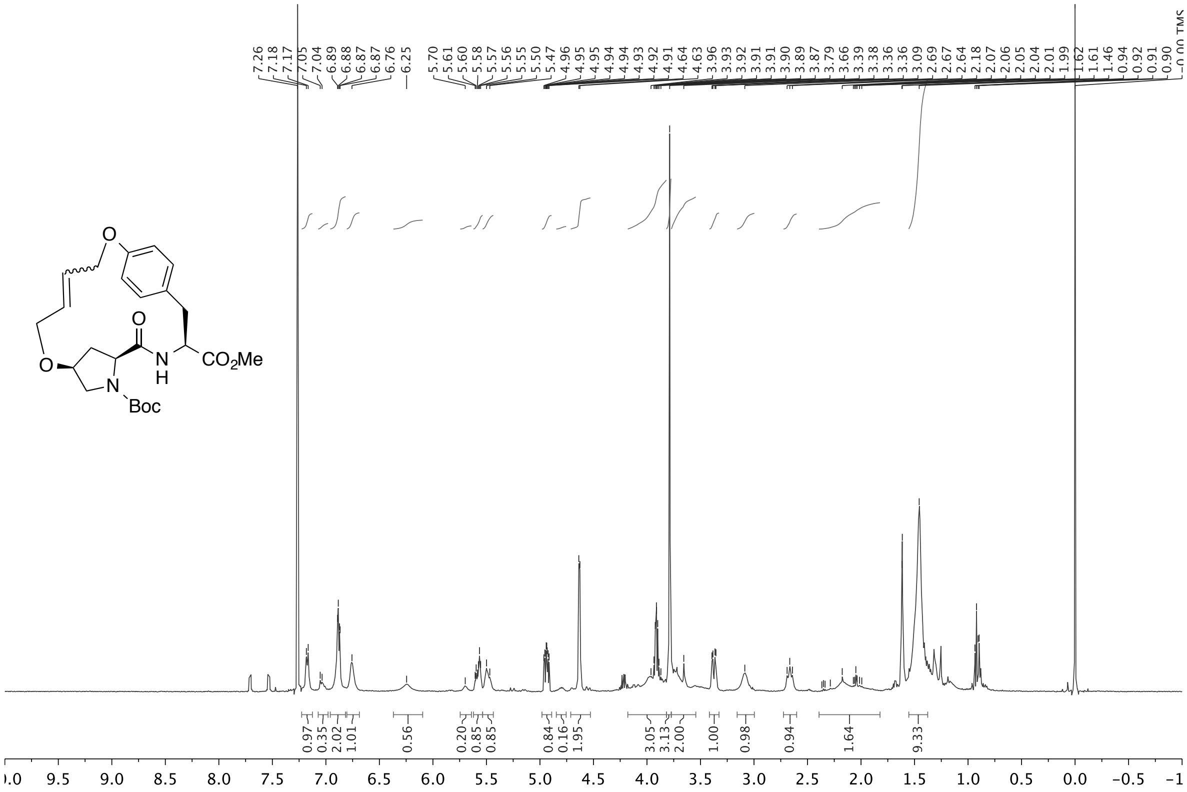

^{1}H NMR of **5’** (500 MHz, $CDCl_3$)

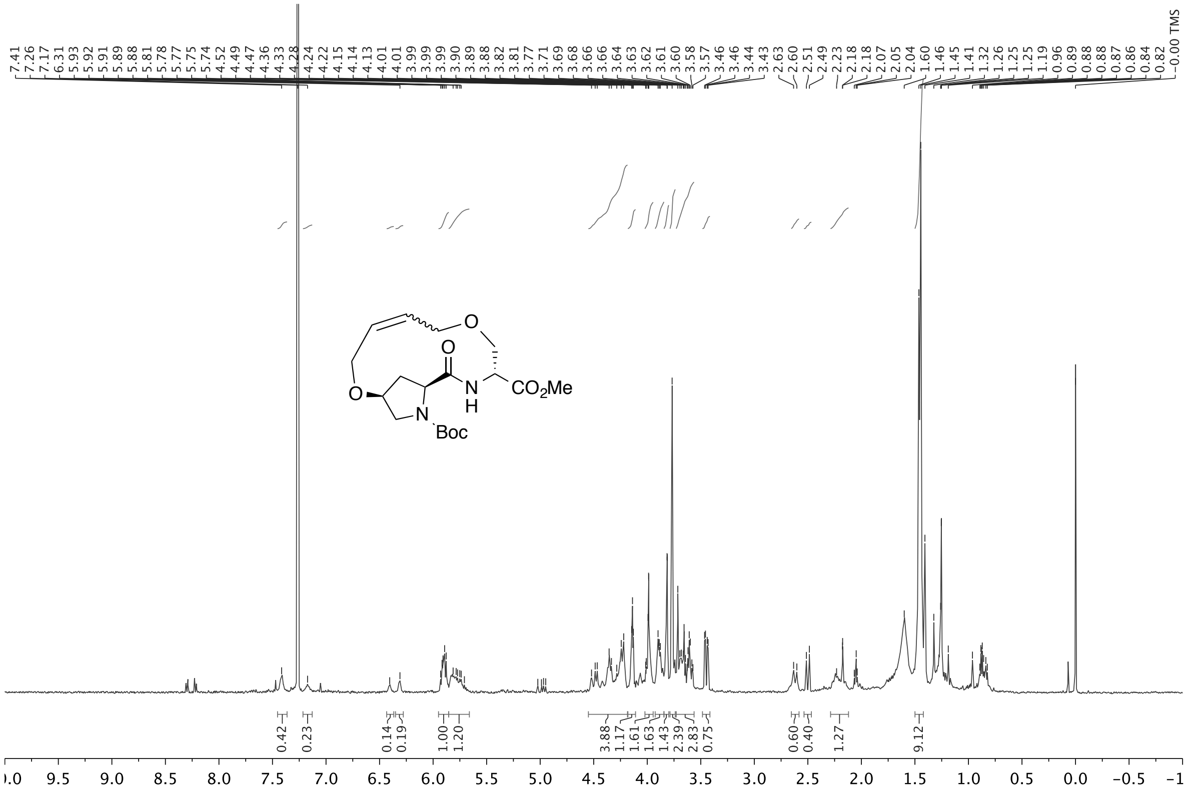

^{1}H NMR of **6’** (500 MHz, $CDCl_3$)

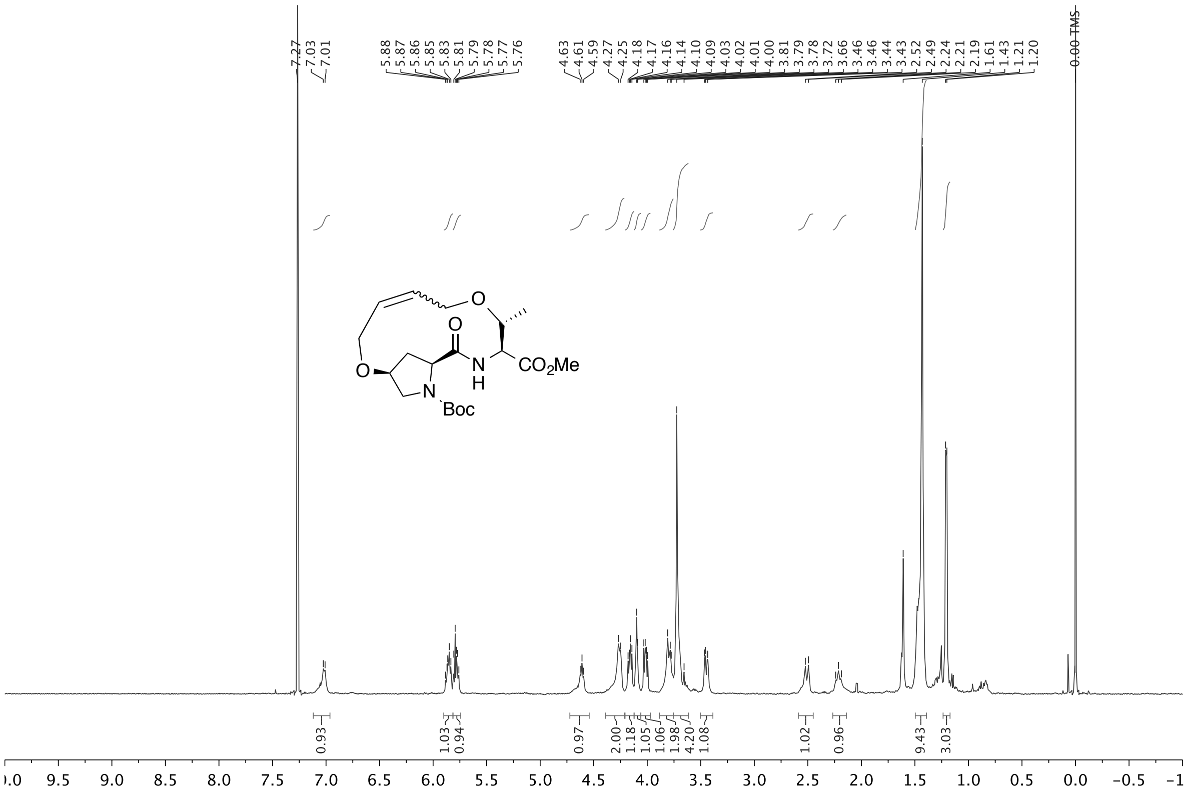

^{1}H NMR of **7’** (500 MHz, $CDCl_3$)

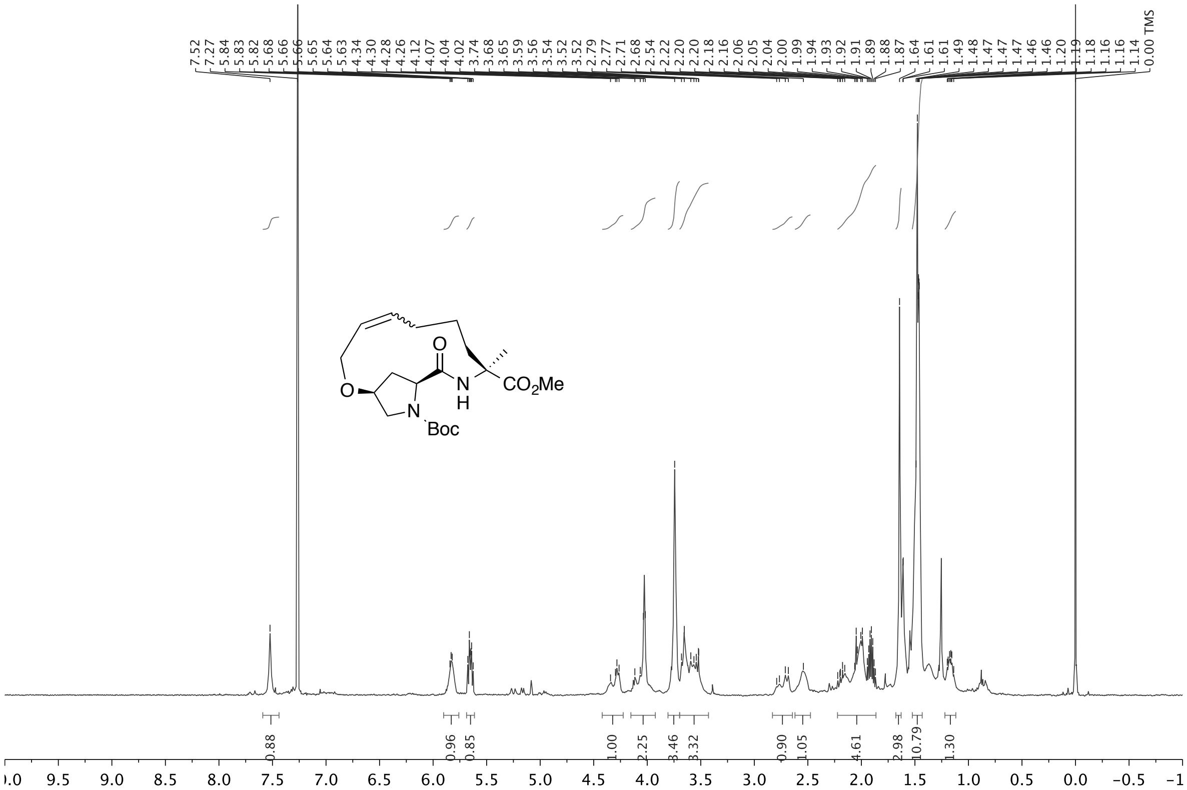

^{1}H NMR of **9** (500 MHz, $CDCl_3$)

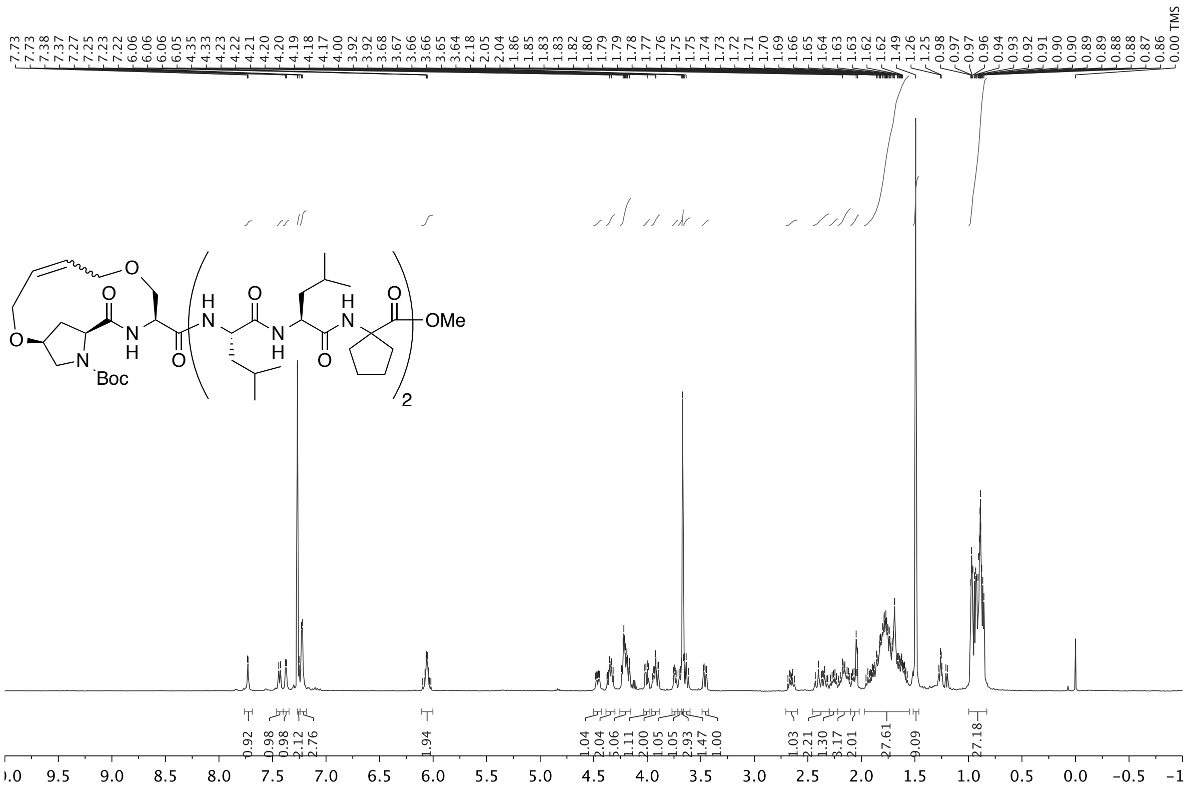

^{1}H NMR of **10** (500 MHz, $CDCl_3$)

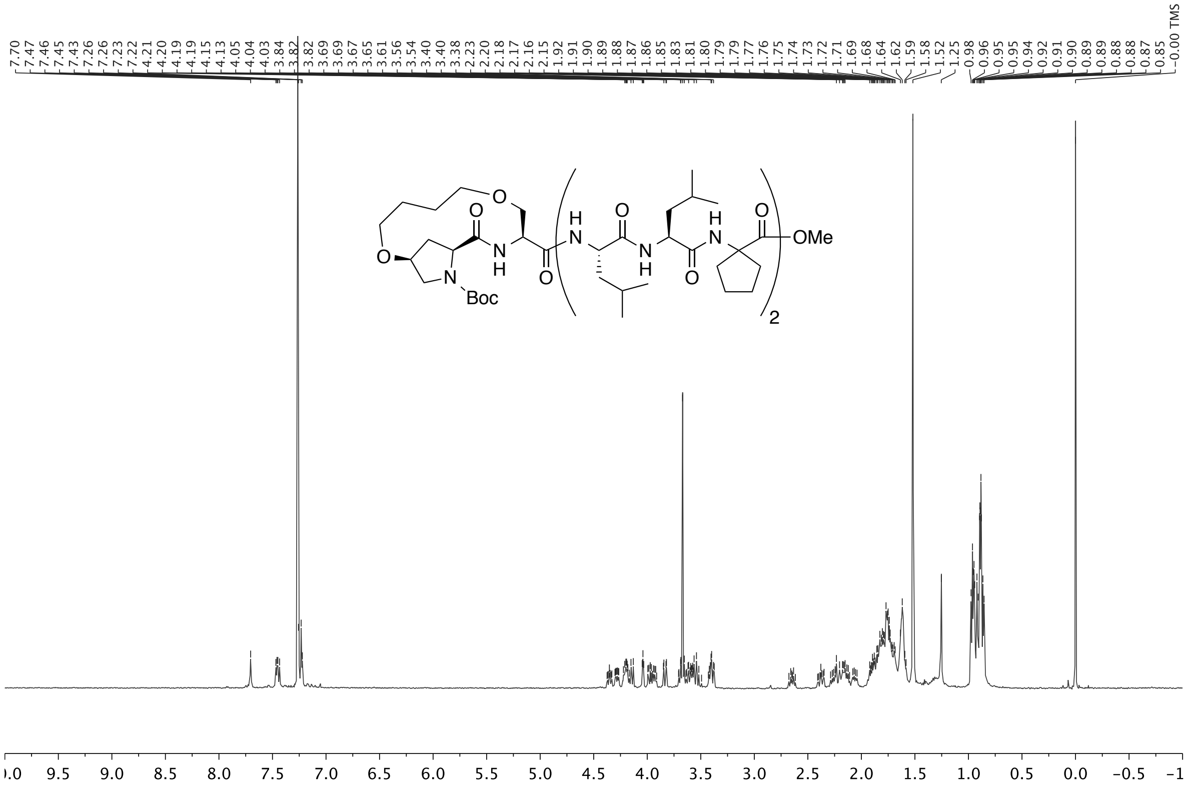

Supplement: Supplementary file 1 [file ijms-22-05364-s001.zip › SI_revised.pdf]
